# Prophylactic proton pump inhibitor use and all-cause mortality in adult sepsis patients: a retrospective analysis based on the MIMIC-IV database

**DOI:** 10.3389/fphar.2025.1545533

**Published:** 2025-06-19

**Authors:** Chendong Ma, Lixin Zhang, Min Wang, Feng Zhang, Lipeng Zhang

**Affiliations:** ^1^ Department of Intensive Care Unit, Inner Mongolia Medical University Affiliated Hospital, Hohhot, China; ^2^ Department of Emergency, The Second Affiliated Hospital of Anhui Medical University, Hefei, Anhui, China

**Keywords:** sepsis, proton pump inhibitors, MIMIC-IV database, risk factors, predictive model, prognosis

## Abstract

**Background:**

Sepsis poses a significant threat to human health, and extensive research has examined the relationship between proton pump inhibitors (PPI) and adverse outcomes in patients with sepsis. However, a consensus on this issue remains elusive. Therefore, this study aims to develop a prognostic model to assess the effectiveness of prophylactic PPI administration in patients with sepsis.

**Methods:**

A retrospective cohort study was conducted using the open-access Medical Information Mart for Intensive Care (MIMIC-IV) database. Patients diagnosed with sepsis according to the Sepsis-3.0 criteria were selected for inclusion. The primary outcome of interest was all-cause mortality occurring between 28 and 90 days following prophylactic PPI use. Secondary outcomes included in-hospital and intensive care unit (ICU) mortality, duration of hospital and ICU stays, and the incidence of adverse events. Stepwise Cox proportional hazards regression analysis was performed, and multivariate Cox regression models were developed and evaluated using receiver operating characteristic (ROC) curves. Additionally, Kaplan–Meier curves were utilized to compare patient survival at 28 and 90 days.

**Results:**

This study included 18,198 sepsis patients. The results demonstrated that prophylactic PPI use was significantly associated with increased 90-day all-cause mortality following ICU admission (*P* < 0.001). Prediction models incorporating 28-day (training AUC 0.74; 95% CI 0.73–0.75) and 90-day (training AUC 0.73; 95% CI 0.72–0.74) outcomes exhibited superior accuracy compared to conventional CCI and SOFA scores. Notably, prophylactic PPI use reduced ICU stay by approximately 1 day in sepsis patients but did not reduce overall hospitalization. Additionally, PPI administration was linked to adverse events including hypoalbuminemia and opportunistic infections.

**Conclusion:**

Prophylactic PPI use failed to improve 28-day or 90-day survival rates in adult sepsis patients. Although PPI use was associated with reduced ICU length of stay, it did not shorten total hospital stay duration. Additionally, PPI administration was linked to clinically significant adverse reactions.

## Introduction

Sepsis, characterized as a life-threatening organ dysfunction resulting from a dysregulated host’s response to infection, represents a significant risk to human life and health, particularly in low- and middle-income countries (Singer et al., 2016; Stephen et al., 2020). Annually, there are an estimated 49 million cases of sepsis worldwide, leading to approximately 11 million fatalities; this accounts for 19.7% of the overall global mortality rate (Rudd et al., 2020). Notwithstanding the recent guidelines issued by the Surviving Sepsis Campaign (Evans et al., 2021), there remains a pressing need for further research into pharmacological interventions and treatment strategies aimed at enhancing the prognosis of sepsis.

It is widely recognized that critically ill patients in intensive care units (ICUs) are susceptible to pressure-related mucosal damage in the gastrointestinal tract, commonly referred to as “pressure-related mucosal injury” (Plummer et al., 2014). Various risk factors contribute to this condition, including sepsis and shock of various origins (Granholm et al., 2019; Zhang et al., 2023). Nevertheless, research on the efficacy of proton pump inhibitors (PPI) in septic patients and their subsequent effects on prognosis is relatively sparse and has yielded inconsistent findings. One study revealed that emergency patients at risk of gastrointestinal bleeding exhibited an increased mortality rate at 90 days post-administration of pantoprazole alongside a reduced number of days’ survival without life support (Marker et al., 2019). Conversely, a meta-analysis suggested that prophylactic treatment for stress could significantly diminish the incidence of clinically significant gastrointestinal bleeding (GI) (relative risk (RR) = 0.58; 95% confidence interval (CI): 0.42–0.81] and overt GI [(RR = 0.48; 95% CI: 0.36–0.63)) (Zhou et al., 2019). In patients not receiving enteral nutrition, the prevention of stress ulcers appears to be advantageous solely in mitigating the risk of overt GI while ineffective in preventing clinically significant GI. Consequently, the prophylactic use of PPI in critically ill patients remains contentious.

Here, we performed a retrospective analysis utilizing the Medical Information Mart for Intensive Care IV (MIMIC-IV 2.2) database to investigate the association between the prophylactic administration of PPI and mortality rates in adult septic patients.

## Materials and methods

### Study design

This is a retrospective study utilizing the MIMIC database. MIMIC is a large, freely available, publicly accessible database that contains deidentified health-related information from patients admitted to the critical care units of the Beth Israel Deaconess Medical Center (BIDMC). The data encompassed in MIMIC-IV, which was gathered from MetaVision bedside monitors, cover the period 2008–2019 (Johnson et al., 2023). It offers comprehensive documentation of various aspects, including patients’ demographic details, laboratory tests results, medication administration, vital signs, surgical interventions, disease diagnoses, medication management, and survival outcomes.

Chendong Ma, the first author of this research, successfully completed the official website CITI course of MIMIC and received associated certification (Record ID 50516983). The data employed in this investigation were sourced from the publicly accessible MIMIC database and was granted ethical approval by the institutional review boards of the Massachusetts Institute of Technology and BIDMC. As a result, obtaining patient consent or additional ethical approval was not considered necessary for the conduct of this study.

### Inclusion and exclusion criteria

Adult patients from the database were screened based on their fulfillment of the diagnostic criteria for sepsis 3.0, which required either a suspected or confirmed infection alongside a sequential organ failure assessment (SOFA) score of two points or higher. The criteria for inclusion were as follows. (1) Patients must have an SOFA score of 2 or higher within 48 h prior to and up to 24 h following the suspected infection or possess a sepsis diagnosis indicated by an ICD code in the discharge summary. (2) Participants must be aged 18 years or older. The exclusion criteria included the following: (1) an ICU stay of less than 24 h; (2) instances of non-first-time data from multiple ICU admissions; (3) prior use of PPI before ICU admission; (4) therapeutic use of PPIs post-admission for conditions such as peptic ulcer disease (including gastric and duodenal ulcers, as well as stress ulcers), upper gastrointestinal bleeding (specifically acute nonvariceal upper gastrointestinal bleeding), various forms of gastritis and esophagitis, gastroesophageal reflux disease, *Helicobacter pylori* infection, and Zollinger–Ellison syndrome.

### Data extraction

In this retrospective study, various parameters were systematically extracted encompassing baseline characteristics such as age, gender, body mass index (BMI), and race. Additionally, vital signs recorded within the first 24 h of admission to ICU were analyzed, including temperature, heart rate, mean arterial pressure (MAP), and respiratory rate. The study also considered comorbidities, including pneumonia, chronic obstructive pulmonary disease (COPD), hypertension, congestive heart failure (CHF), myocardial infarction (MI), renal disease, liver disease, diarrhea, osteoporosis, diabetes, and cancer.

Laboratory test indices were evaluated, comprising white blood cell count (WBC), percentage of neutrophils (N%), percentage of lymphocytes (L%), hemoglobin levels, platelet count (PLT), creatinine, blood urea nitrogen (BUN), sodium ions, potassium ions, chloride ions, prothrombin time (PT), activated partial thromboplastin time (APTT), international normalized ratio (INR), lactate levels, pulse oxygen saturation (SpO_2_), pH, partial pressure of oxygen (PO_2_), partial pressure of carbon dioxide (PCO_2_), bicarbonate ions, and blood glucose levels.

Possibly related risk factors were identified, including mechanical ventilation (MV) exceeding 48 h, coagulation disorders (defined as INR > 1.5, PLT < 50 × 10^9^/L or APTT > twice the normal value), craniocerebral and cervical spinal cord injuries, acute kidney injury (AKI), chronic liver disease or acute liver failure, shock, cardiovascular and cerebrovascular diseases, ICU length of stay (LOS) greater than 7 days, and the use of glucocorticoids, non-selective nonsteroidal anti-inflammatory drugs (non-selective NSAIDs), and Cox-2 selective NSAIDs.

Adverse reactions were also documented, including hypomagnesemia, vitamin B12 deficiency, hypoalbuminemia, and positive tests for *Clostridium difficile* in fecal samples. Furthermore, organ function support measures such as MV, renal replacement therapy (RRT), and extracorporeal membrane oxygenation (ECMO) were assessed. Disease severity was evaluated using scores such as the SOFA, simplified acute physiology score (SAPS II), and Charlson comorbidities index (CCI).

These indicators reflect the average levels recorded within the first 24 h of ICU admission, while adverse reactions were determined based on the lowest values or positive test results observed during ICU stay following the use of PPIs. The code utilized for data extraction is accessible on GitHub (https://github.com/MIT-LCP/mimic-iv).

### Outcomes

The primary outcome of the study was all-cause mortality occurring 28–90 days following the administration of prophylactic PPIs. The secondary outcomes assessed included mortality during hospitalization, mortality within the ICU, duration of hospital stay, duration of ICU stay, and the occurrence of adverse reactions.

### Statistical analysis

The data were systematically organized and analyzed. Missing values were addressed using the expectation maximization algorithm for interpolation when the proportion of missing data was less than 20%, while direct deletion was employed for instances where missing data exceeded 20%. The normality of continuous variables was evaluated using the Shapiro–Wilk test. Data exhibiting a normal distribution were reported as mean ± SD, with independent sample T-tests utilized for analysis. Conversely, non-normally distributed data were presented as M (P25, P75) and analyzed using the Mann–Whitney U test. Categorical variables were represented as counts (n) and percentages (%), with analyses conducted using the chi-square test (*χ* ^*2*^) or Fisher’s exact test. The Bonferroni correction was applied to account for multiple comparisons of rates or component ratios.

The baseline demographic characteristics were evaluated, and then the propensity score matching method was used to match the subjects in a 1:1 ratio. Stepwise regression analyses were conducted to assess the 28- and 90-day survival outcomes of patients with sepsis, distinguishing between survivors and non-survivors. In order to develop a prognostic model, variables that demonstrated statistical significance (*P* < 0.05) were selected for single-factor receiver operating characteristic (ROC) curve analysis and collinearity analysis. Following this, a nomogram model was established: the 28-day model incorporated the variables of age, ICU LOS, SOFA, CCI, shock, AKI, MV, lactate, INR, lymphocytes, and creatinine. The 90-day model included age, ICU LOS, PPI, SOFA, CCI, shock, AKI, MV, lactate, INR, lymphocytes, and creatinine. The models were represented by nomogram, and ROC curves were generated to assess sensitivity, specificity, and calibration. Additionally, Kaplan–Meier survival analysis was performed utilizing the log-rank test to investigate the association between the identified factors and survival time and outcomes.

The possible adverse effects associated with the prophylactic use of PPI, including electrolyte imbalances (notably hypomagnesemia), nutrient deficiencies (specifically vitamin B_12_ malabsorption), hypoalbuminemia, and the risk of opportunistic infections (such as *C. difficile*), were illustrated utilizing bar charts, box plots, and cluster plots. Furthermore, various categories of PPIs and their respective administration methods were represented through mixed charts and correspondence analysis.

In order to further analyze the interaction between the prophylactic use of PPI and confounding factors on all-cause mortality in sepsis, a subgroup analysis of related factors was performed, and the results were displayed in forest plots.

The research employed SPSS version 26.0 software for statistical analysis, while GraphPad Prism version 10.2.1 and R Studio version 2023.06.2 + 561 were utilized for data visualization. Statistical significance was defined as a two-sided *P* value less than 0.05.

## Results

### Demographic characteristics

**FIGURE 1:**
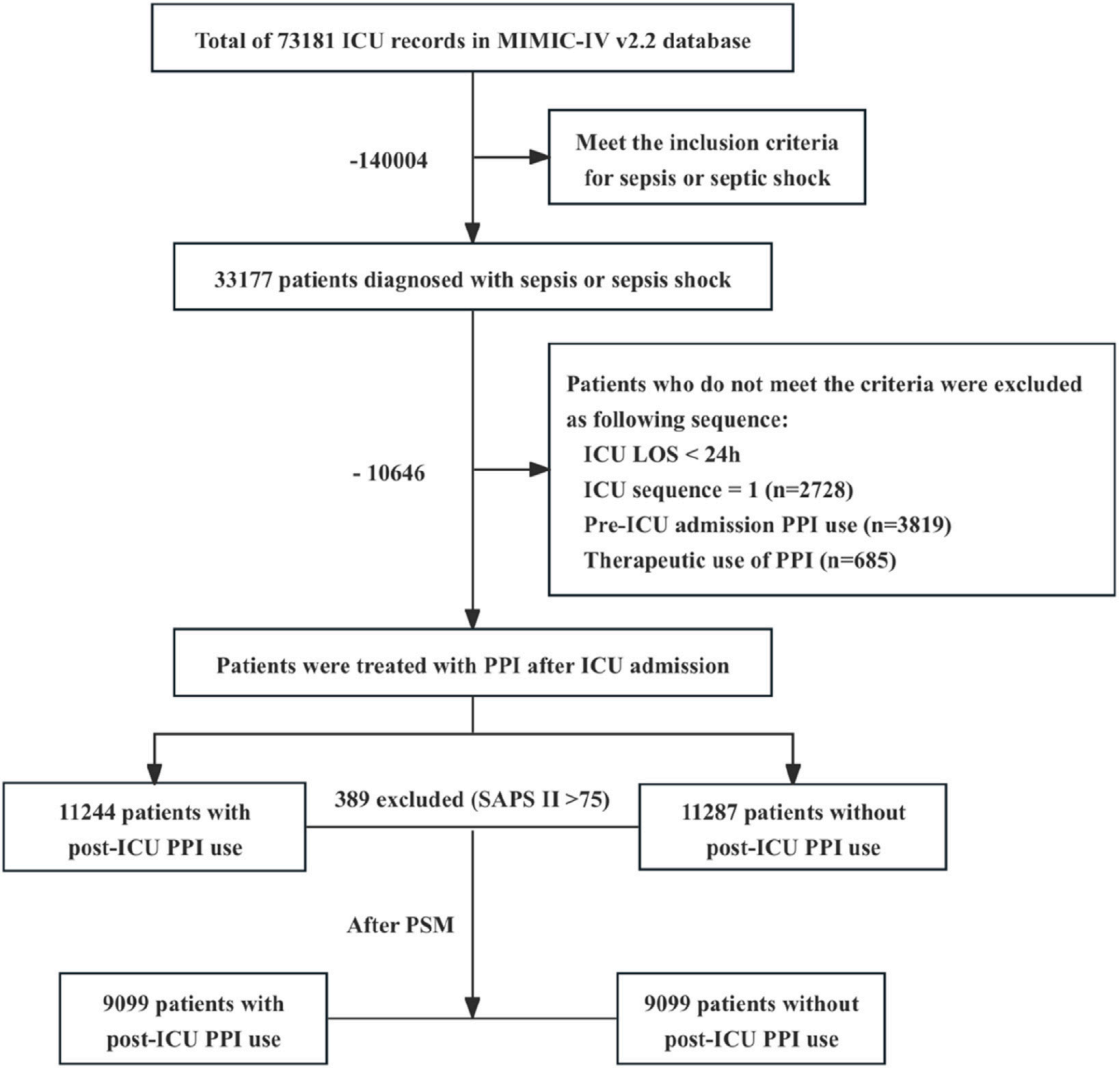
Flowchart illustrating the process of cohort selection. ICU, intensive care unit; MIMIC-IV, Medical Information Mart for Intensive Care IV; LOS, length of stay; PPI, proton pump inhibitor. Therapeutic applications of PPI encompass the following conditions: peptic ulcers, which include gastric ulcers, duodenal ulcers, and stress ulcers (n = 684); upper gastrointestinal bleeding, specifically acute nonvariceal upper gastrointestinal bleeding (n = 1); various forms of gastritis and esophagitis (n = 0); gastroesophageal reflux disease (n = 0); *Helicobacter pylori* infection (n = 0); Zollinger–Ellison syndrome (n = 0).

In the MIMIC-IV database, a cohort of 33,177 adult patients was identified which satisfied the diagnostic criteria for sepsis or septic shock as defined by Sepsis 3.0. Following a rigorous exclusion process, a total of 22,531 patients were ultimately included in this study (Figure 1). The patients were categorized into two groups: the post-ICU PPI user group (“Users”), comprising 11,244 individuals, and the post-ICU non-PPI user group (“Non-users”), comprising 11,287 individuals. The baseline characteristics and clinical data of the patients initially included in the study are presented in Table 1.

**TABLE 1:** Baseline demographic and clinical attributes of sepsis patients.

Variables	All (n = 22,531)	Post-ICU proton pump inhibitor use		*P*
		Non-users (n = 11,287)	Users (n = 11,244)	
Patient characteristics				
Age (yr)	67.8 (56.5,78.7)	67.8 (56.2, 78.8)	67.7 (56.7, 78.7)	0.524
Sex (male), n (%)	13,081 (58.1)	6,673 (59.1)	6,408 (57.0)	0.001
BMI (kg/m^2^)	27.9 (23.6, 31.7)	27.3 (23.6, 31.6)	27.5 (23.7, 31.8)	0.033
Race, n (%)				
Asian	632 (2.8)	345 (3.1)	287 (2.6)	0.002
Black	2,260 (10.0)	1,094 (9.7)	1,166 (10.4)	
Hispanic	790 (3.5)	392 (3.5)	398 (3.5)	
White	15,188 (67.4)	7,539 (66.8)	7,649 (68.0)	
Other	3,661 (16.2)	1,917 (17.0)	1,744 (15.5)	
Vital signs, M (P_25_, P_75_)				
Temperature (°C)	36.9 (36.6, 37.2)	36.9 (36.6, 37.2)	36.9 (36.6, 37.2)	0.262
Heart rate (bpm)	85.4 (75.4, 97.4)	84.0 (74.9, 95.7)	86.9 (76.2, 99.0)	<0.001
MAP (mmHg)	75.3 (69.7, 82.1)	75.7 (70.3, 82.2)	74.8 (69.2,81.9)	<0.001
Respiratory rate (rpm)	19.1 (16.8, 22.0)	18.8 (16.7, 21.6)	19.4 (16.9, 22.4)	<0.001
Laboratory indexes				
WBC (×10^9^/L)	11.7 (8.5, 15.8)	11.9 (8.8, 15.7)	11.5 (8.1, 16.0)	<0.001
Neutrophils %	79.5 (75.6, 85.2)	79.0 (75.6, 84.3)	80.0 (75.5, 86.1)	<0.001
Lymphocytes %	11.7 (7.0, 15.4)	12.5 (8.0, 16.0)	10.8 (6.2, 14.7)	<0.001
Hemoglobin (g/dL)	10.5 (9.1, 11.9)	10.7 (9.5, 12.2)	10.1 (8.8, 11.7)	<0.001
Platelets (×10^9^/L)	183.0 (130.5, 249.5)	183.0 (136.0, 244.5)	183.0 (122.7, 255.3)	0.007
Creatinine (mg/dL)	1.05 (0.8, 1.7)	1.0 (0.8, 1.5)	1.2 (0.8, 1.9)	<0.001
BUN (mg/dL)	21.0 (14.3, 35.5)	19.0 (13.3, 30.5)	24.0 (15.5, 40.7)	<0.001
Sodium (mmol/L)	138.5 (136.0, 141.0)	138.7 (136.3, 141.0)	138.5 (135.5, 141.0)	<0.001
Potassium (mmol/L)	4.2 (3.9, 4.6)	4.2 (3.9, 4.6)	4.2 (3.8, 4.6)	0.345
Chloride (mmol/L)	104.6 (100.5, 108.0)	105.0 (101.3, 108.3)	104.0 (99.5, 108.0)	<0.001
PT (s)	14.4 (12.8, 16.9)	14.1 (12.7, 16.1)	14.8 (12.9, 18.0)	<0.001
APTT (s)	32.2 (27.9, 38.7)	31.4 (27.6, 37.0)	33.1 (28.3, 40.7)	<0.001
INR	1.3 (1.2, 1.6)	1.3 (1.2, 1.5)	1.3 (1.2, 1.7)	<0.001
Lactate (mmol/L)	1.9 (1.4, 2.6)	1.9 (1.4, 2.5)	1.9 (1.4, 2.8)	<0.001
SpO_2_ (%)	97.3 (95.8, 98.6)	97.4 (96.0, 98.6)	97.2 (95.7, 98.5)	<0.001
PH	7.4 (7.3, 7.4)	7.4 (7.3, 7.4)	7.4 (7.3, 7.4)	<0.001
PO_2_ (mmHg)	124.0 (78.7, 180.0)	137.0 (84.7, 207.9)	113.0 (73.3, 159.3)	<0.001
PCO_2_ (mmHg)	41.3 (37.0, 46.0)	41.3 (37.4, 45.8)	41.3 (36.5, 46.7)	0.510
Bicarbonate (mmol/L)	23.0 (20.0, 25.1)	23.0 (20.7, 25.0)	22.5 (19.5, 25.3)	<0.001
Glucose (mg/dL)	130.0 (109.0, 163.0)	128.0 (108.5, 157.0)	132.0 (109.3, 169.5)	<0.001
Comorbidities, n (%)				
Pneumonia	5,267 (23.4)	2,294 (20.3)	2,973 (26.4)	<0.001
COPD	2,795 (12.4)	1,154 (10.2)	1,641 (14.6)	<0.001
Hypertension	14,566 (64.6)	7,291 (64.6)	7,275 (64.7)	0.869
CHF	7,203 (32.0)	3,288 (29.1)	3,915 (34.8)	<0.001
MI	4,002 (17.8)	1,890 (16.7)	2,112 (18.8)	<0.001
Renal disease	5,460 (24.2)	2,351 (20.8)	3,109 (27.7)	<0.001
Liver disease	3,300 (14.6)	963 (8.5)	2,337 (20.8)	<0.001
Osteoporosis	1,046 (4.6)	505 (4.5)	541 (4.8)	0.229
Diabetes	7,129 (31.6)	3,385 (30.0)	3,744 (33.3)	<0.001
Cancer	2,905 (12.9)	1,305 (11.6)	1,600 (14.2)	<0.001
Organ support, n (%)				
Mechanical ventilation	12,382 (55.0)	5,928 (52.5)	6,454 (57.4)	<0.001
Vasopressors	11,597 (51.5)	5,751 (51.0)	5,846 (52.0)	0.118
RRT	2,181 (9.7)	676 (6.0)	1,505 (13.4)	<0.001
ECMO	56 (0.2)	9 (0.1)	47 (0.4)	<0.001
Severity score				
SOFA	4.2 (2.7, 6.4)	3.9 (2.6, 5.9)	4.6 (2.8, 6.9)	<0.001
SAPS II	38.0 (30.0, 48.0)	36.0 (29.0, 46.0)	40.0 (31.0, 49.0)	<0.001
CCI	6.0 (4.0, 8.0)	5.0 (4.0, 7.0)	6.0 (4.0, 8.0)	<0.001
Outcomes				
28-day mortality	4,179 (18.5)	1,846 (16.4)	2,333 (20.7)	<0.001
90-day mortality	5,741 (25.5)	2,412 (21.4)	3,329 (29.6)	<0.001
In-hospital mortality	3,344 (14.8)	1,429 (12.7)	1,915 (17.0)	<0.001
ICU mortality	2,071 (9.2)	889 (7.9)	1,182 (10.5)	<0.001
Hospital LOS days	8.3 (5.2, 14.4)	7.4 (4.9, 12.2)	9.5 (5.7, 16.7)	<0.001
ICU LOS days	3.1 (1.9, 6.2)	2.7 (1.7, 5.2)	3.7 (2.0, 7.4)	<0.001

BMI, body mass index; MAP, mean arterial pressure; WBC, white blood cell; BUN, blood urea nitrogen; PT, prothrombin time; APTT, activated partial thromboplastin time; INR, international standard ratio; SpO_2_, pulse oxygen saturation; PO_2_, partial pressure of oxygen; PCO_2_, partial pressure of carbon dioxide; COPD, chronic obstructive pulmonary disease; CHF, congestive heart failure; MI, myocardial infarction; RRT, renal replacement therapy; ECMO, extracorporeal membrane oxygenation; SOFA, sequential organ failure assessment; SAPA II, simplified acute physiology score; CCI, Charlson comorbidities index; Hospital LOS days, hospital length of stay days; ICU LOS days, intensive care unit length of stay days.

As presented in Table 1, there were statistically significant differences in patient characteristics, vital signs, laboratory parameters, complications, risk factors, adverse reactions, organ support, disease severity scores, and prognosis between the Users and Non-users groups (*P* < 0.05). Those patients utilizing PPIs exhibited a higher prevalence of complications such as pneumonia, COPD, CFH, MI, renal disease, hepatic disease, diabetes, and cancer, suggesting a range of complex clinical conditions within this cohort. Furthermore, with respect to organ support and disease severity scores, the PPI users demonstrated a significantly greater proportion of patients requiring MV, RRT, and ECMO compared to their non-PPI counterparts (*P* < 0.001). Additionally, the SOFA, SAPS II, and CCI scores indicated an increased risk of complexity and severity of patient conditions among the PPI users.

### Demographic characteristics after propensity score matching

**FIGURE 2:**
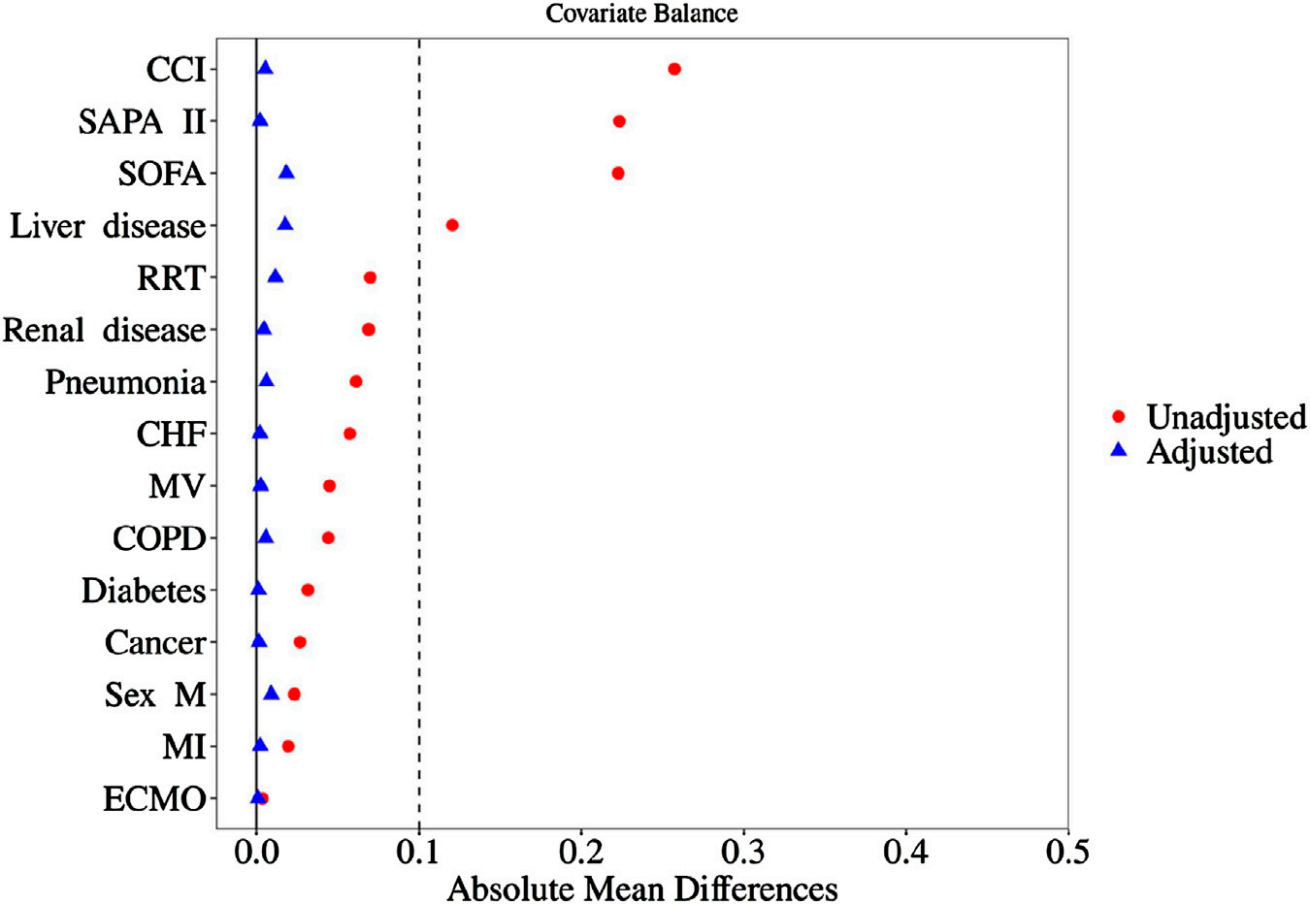
SMD analysis diagram before and after matching. SOFA, sequential organ failure assessment; SAPA II, simplified acute physiology score; CCI, Charlson comorbidities index; RRT, renal replacement therapy; CHF, congestive heart failure; MV, mechanical ventilation; COPD, chronic obstructive pulmonary disease; MI, myocardial infarction; ECMO, extracorporeal membrane.

As shown in Table 1, baseline indicators such as disease severity, past comorbidities, and organ support needs were significantly higher in the Users than in the Non-users group (all *P* < 0.05). In order to control baseline confounding, the study first excluded 389 high-risk patients with a SAPS II score > 75 and then used propensity score matching to perform 1:1 inter-group matching. After this, there were still statistical differences in previous liver disease (*P* < 0.001) and RRT treatment rate (*P* = 0.003) between the two groups, but the standardized mean difference (SMD) was <0.05. According to the SMD threshold (<0.1 indicates balance), the baseline characteristics of the matched data were balanced and comparable. The baseline characteristics and clinical data of patients before and after matching are shown in Table 2, and the results of SMD analysis are shown in Figure 2.

**TABLE 2:** Propensity score-matched analysis of baseline characteristics in sepsis.

Variables, M (Q_1_, Q_3_) or n (%)	Before PSM				After PSM			
	Non-users (n_1_ = 11,144)	Users (n_2_ = 10,998)	*P*	SMD	Non-users (n_3_ = 9,099)	Users (n_4_ = 9,099)	*P*	SMD
SOFA	3.9 (2.6, 5.8)	4.5 (2.8, 6.8)	<0.001	0.223	4.1 (2.8, 6.1)	4.0 (2.7, 6.3)	0.578	0.019
SAPA II	36.0 (29.0, 45.0)	39.0 (31.0, 49.0)	<0.001	0.224	38.0 (31.0, 47.0)	39.0 (31.0, 47.0)	0.591	−0.002
CCI	5.0 (4.0, 7.0)	6.0 (4.0, 8.0)	<0.001	0.257	6.0 (4.0, 8.0)	6.0 (4.0, 8.0)	0.466	0.005
Sex			<0.001				0.220	
F	4,547 (40.8)	4,744 (43.1)		0.047	3,998 (43.9)	3,916 (43.0)		−0.018
M	6,597 (59.2)	6,254 (56.9)		−0.047	5,101 (56.1)	5,183 (57.0)		0.018
CHF			<0.001				0.766	
0	7,909 (71.0)	7,175 (65.2)		−0.120	6,046 (66.5)	6,027 (66.3)		−0.004
1	3,235 (29.0)	3,823 (34.8)		0.120	3,053 (33.6)	3,072 (33.8)		0.004
Diabetes			<0.001				0.874	
0	7,804 (70.0)	7,355 (66.9)		−0.067	6,152 (67.6)	6,142 (67.5)		−0.002
1	3,340 (30.0)	3,643 (33.1)		0.067	2,947 (32.4)	2,957 (32.5)		0.002
Liver disease			<0.001				<0.001	
0	10,206 (91.6)	8,747 (79.5)		−0.299	8,161 (89.7)	8,001 (87.9)		−0.054
1	938 (8.4)	2,251 (20.5)		0.299	938 (10.3)	1,098 (12.1)		0.054
Renal disease			<0.001				0.481	
0	8,849 (79.4)	7,975 (72.5)		−0.154	6,872 (75.5)	6,831 (75.1)		−0.010
1	2,295 (20.6)	3,023 (27.5)		0.154	2,227 (24.5)	2,268 (24.9)		0.010
Cancer			<0.001				0.777	
0	9,883 (88.7)	9,460 (86.0)		−0.077	7,892 (86.7)	7,879 (86.6)		−0.004
1	1,261 (11.3)	1,538 (14.0)		0.077	1,207 (13.3)	1,220 (13.4)		0.004
MV			<0.001				0.732	
0	5,334 (47.9)	4,772 (43.4)		−0.090	4,099 (45.1)	4,122 (45.3)		0.005
1	5,810 (52.1)	6,226 (56.6)		0.090	5,000 (55.0)	4,977 (54.7)		−0.005
RRT			<0.001				0.003	
0	10,497 (94.2)	9,590 (87.2)		−0.209	8,452 (92.9)	8,347 (91.7)		−0.042
1	647 (5.8)	1,408 (12.8)		0.209	647 (7.1)	752 (8.3)		0.042
COPD			<0.001				0.246	
0	10,009 (89.9)	9,391 (85.4)		−0.125	7,974 (87.6)	7,922 (87.1)		−0.017
1	1,131 (10.1)	1,602 (14.6)		0.125	1,125 (12.4)	1,177 (12.9)		0.017
MI			<0.001				0.700	
0	9,287 (83.3)	8,952 (81.4)		−0.050	7,462 (82.0)	7,442 (81.8)		−0.006
1	1,857 (16.7)	2,046 (18.6)		0.050	1,637 (18.0)	1,657 (18.2)		0.006
Pneumonia			<0.001				0.340	
0	8,889 (79.8)	8,097 (73.7)		−0.139	6,934 (76.2)	6,879 (75.6)		−0.014
1	2,251 (20.2)	2,896 (26.3)		0.139	2,165 (23.8)	2,220 (24.4)		0.014
ECMO			<0.001				0.088	
0	11,137 (99.9)	10,953 (99.6)		−0.054	9,092 (99.9)	9,084 (99.8)		−0.022
1	7 (0.1)	45 (0.4)		0.054	7 (0.1)	15 (0.2)		0.022

SOFA, sequential organ failure assessment; SAPA II, simplified acute physiology score; CCI, Charlson comorbidities index; CHF, congestive heart failure; MV, mechanical ventilation; RRT, renal replacement therapy; COPD, chronic obstructive pulmonary disease; MI, myocardial infarction; ECMO, extracorporeal membrane oxygenation.

### 28-day and 90-day survivors and non-survivors

The baseline characteristics of the groups categorized by the 28- and 90-day ICU survival and non-survival groups are detailed in Tables 3, 4. The study finally encompassed a total of 18,198 patients diagnosed with sepsis. Within the 28-day observation period, 14,922 patients (82%) were classified as survivors, whereas 3,276 (18%) were identified as non-survivors. At the 90-day mark, survival rates were recorded at 13,623 patients (74.9%) for survivors and 4,575 (25.1%) for non-survivors. The mortality rate exhibited an increase of 7.1% over the subsequent 60-day observation period, which may be attributed to factors such as advanced age, a greater number of comorbidities, an increased requirement for organ support, and elevated disease severity scores among non-survivors (*P* < 0.001). The baseline characteristics and clinical data of sepsis patients in the Survival and Non-survival groups at different times are shown in Tables 3, 4.

**TABLE 3:** Baseline demographic and clinical characteristics of sepsis patients between 28-day survivors and non-survivors.

Variables	All (n = 18,198)	Survival (n = 14,922)	Non-survival (n = 3,276)	*P*
Patient characteristics				
Age (yr)	67.8 (56.5, 78.7)	67.7 (56.7, 78.1)	75.4 (63.0, 84.7)	<0.001
Sex (male), n (%)	10,284 (56.5)	8,480 (56.8)	1,804 (55.1)	0.066
BMI (kg/m^2^)	27.3 (23.6, 31.7)	27.6 (23.8, 32.0)	26.1 (22.4, 30.3)	<0.001
Laboratory index				
WBC (×10^9^/L)	11.7 (8.5, 15.8)	11.5 (8.4, 15.5)	12.5 (8.9, 17.4)	<0.001
Neutrophils (%)	80.0 (76.0, 86.0)	79.0 (76.0, 85.0)	81.0 (76.0, 88.0)	<0.001
Lymphocytes (%)	12.0 (7.0, 15.0)	12.0 (7.0, 16.0)	9.0 (5.0, 13.0)	<0.001
Hemoglobin (g/dL)	10.5 (9.2, 11.9)	10.5 (9.2, 11.9)	10.2 (8.9, 11.9)	<0.001
Platelet (×10^9^/L)	187.0 (134.5, 253.5)	186.0 (135.3, 250.0)	192.4 (128.1, 268.3)	0.229
Creatinine (mg/dL)	1.1 (0.8, 1.6)	1.0 (0.8, 1.5)	1.3 (0.9, 2.1)	<0.001
BUN (mg/dL)	21.4 (14.5, 35.0)	20.0 (14.0, 32.3)	29.3 (18.9, 47.1)	<0.001
Sodium (mmol/L)	138.7 (136.0, 141.0)	138.7 (136.0, 141.0)	138.8 (135.5, 142.0)	0.015
Potassium (mmol/L)	4.2 (3.9, 4.6)	4.2 (3.9, 4.6)	4.2 (3.9, 4.7)	<0.001
Chloride (mmol/L)	104.5 (100.5, 108.2)	104.8 (101.0, 108.3)	103.5 (99.0, 107.8)	<0.001
PT (s)	14.3 (12.7, 16.7)	14.2 (12.7, 16.3)	15.3 (13.1, 19.6)	<0.001
APTT (s)	32.0 (27.8, 38.4)	31.8 (27.7, 37.7)	33.7 (28.2, 43.4)	<0.001
INR	1.3 (1.2, 1.5)	1.3 (1.2, 1.5)	1.4 (1.2, 1.8)	<0.001
Lactate (mmol/L)	1.9 (1.4, 2.6)	1.8 (1.3, 2.5)	2.1 (1.5, 3.1)	<0.001
SpO_2_ (%)	97.3 (95.8, 98.6)	97.3 (95.9, 98.6)	97.0 (95.2, 98.5)	<0.001
PH	7.4 (7.3, 7.4)	7.4 (7.3, 7.4)	7.4 (7.3, 7.4)	<0.001
Bicarbonate (mmHg)	23.0 (20.3, 25.3)	13.0 (20.5, 25.4)	22.0 (18.8, 25.0)	<0.001
Glucose (mg/dl)	130.2 (109.0, 163.0)	129.0 (109.0, 160.0)	138.5 (111.8, 179.0)	<0.001
Comorbidities, n (%)				
Pneumonia	4,385 (24.1)	3,288 (22.0)	1,097 (33.5)	<0.001
COPD	2,302 (12.6)	1,822 (12.2)	480 (14.7)	<0.001
Hypertension	12,075 (66.4)	9,882 (66.2)	2,193 (66.9)	0.432
CHF	6,125 (33.7)	4,826 (32.3)	1,299 (39.7)	<0.001
MI	3,294 (18.1)	2,587 (17.3)	707 (21.6)	<0.001
Renal disease	4,495 (24.7)	3,533 (23.7)	962 (29.4)	<0.001
Liver disease	2,036 (11.2)	1,540 (10.3)	496 (15.1)	<0.001
Osteoporosis	908 (5.0)	747 (5.0)	161 (4.9)	0.828
Diabetes	5,904 (32.4)	4,874 (32.7)	1,030 (31.4)	0.176
Cancer	2,427 (13.3)	1,735 (11.6)	692 (21.1)	<0.001
Organ support, n (%)				
Mechanical ventilation	9,977 (54.8)	7,929 (53.1)	2,048 (62.5)	<0.001
Vasopressors	9,218 (50.7)	7,257 (48.6)	1,961 (59.9)	<0.001
RRT	1,399 (7.7)	1,002 (6.7)	397 (12.1)	<0.001
ECMO	22 (0.1)	13 (0.1)	9 (0.3)	0.012
Severity score				
SOFA	4.1 (2.7, 6.2)	4.0 (2.6, 5.9)	5.0 (3.1, 7.4)	<0.001
SAPS II	38.0 (31.0, 47.0)	37.0 (29.0, 45.0)	47.0 (38.0, 56.0)	<0.001
CCI	6.0 (4.0, 8.0)	6.0 (4.0, 8.0)	7.0 (5.0, 9.0)	<0.001
Outcomes				
Hospital LOS days	8.3 (5.3, 14.2)	8.7 (5.6, 15.0)	6.9 (3.7, 11.9)	<0.001
ICU LOS days	3.1 (1.9, 6.2)	3.0 (1.8, 6.0)	4.1 (2.2, 7.4)	<0.001

BMI, body mass index; WBC, white blood cell; BUN, blood urea nitrogen; PT, prothrombin time; APTT, activated partial thromboplastin time; INR, international standard ratio; SpO_2_, pulse oxygen saturation; COPD, chronic obstructive pulmonary disease; CHF, congestive heart failure; MI, myocardial infarction; RRT, renal replacement therapy; ECMO, extracorporeal membrane oxygenation; SOFA, sequential organ failure assessment; SAPA II, simplified acute physiology score; CCI, Charlson comorbidities index; hospital LOS days, hospital length of stay days; ICU LOS days, intensive care unit length of stay days.

**TABLE 4:** Baseline demographic and clinical characteristics of sepsis patients between 90-day survivors and non-survivors.

Variables	All (n = 18,198)	Survival (n = 13,623)	Non-survival (n = 4,575)	*P*
Patient characteristics				
Age (yr)	67.8 (56.5, 78.7)	67.1 (56.1, 77.5)	75.1 (63.5, 84.6)	<0.001
Sex (male), n (%)	10,284 (56.5)	7,754 (56.9)	2,530 (55.3)	0.056
BMI (kg/m^2^)	27.3 (23.6, 31.7)	27.7 (24.0, 32.1)	26.1 (22.4, 30.2)	<0.001
Laboratory index				
WBC (×10^9^/L)	11.7 (8.5, 15.8)	11.5 (8.5, 15.5)	12.1 (8.6, 16.8)	<0.001
Neutrophils %	80.0 (76.0, 86.0)	79.0 (76.0, 85.0)	81.0 (76.0, 87.0)	<0.001
Lymphocytes %	12.0 (7.0, 15.0)	12.0 (8.0, 16.0)	10.0 (5.0, 13.0)	<0.001
Hemoglobin (g/dL)	10.5 (9.2, 11.9)	10.5 (9.3, 12.0)	10.1 (8.8, 11.7)	<0.001
Platelet (g/dL)	187.0 (134.5, 253.5)	185.0 (135.3, 248.0)	193.7 (130.0, 269.5)	0.003
Creatinine (mg/dL)	1.1 (0.8, 1.6)	1.0 (0.8, 1.5)	1.3 (0.9, 2.1)	<0.001
BUN (mg/dL)	21.4 (14.5, 35.0)	19.5 (13.5, 31.0)	29.0 (18.7, 46.7)	<0.001
Sodium (mmol/L)	138.7 (136.0, 141.0)	138.7 (136.0, 141.0)	138.7 (135.3, 142.0)	0.291
Potassium (mmol/L)	4.2 (3.9, 4.6)	4.2 (3.9, 4.6)	4.2 (3.8, 4.7)	<0.001
Chloride (mmol/L)	104.5 (100.5, 108.2)	105.0 (101.0, 108.3)	103.4 (99.0, 107.7)	<0.001
PT (s)	14.3 (12.7, 16.7)	14.1 (12.6, 16.1)	15.2 (13.0, 19.3)	<0.001
APTT (s)	32.0 (27.8, 38.4)	31.6 (27.7, 37.5)	33.4 (28.2, 42.1)	<0.001
INR	1.3 (1.2, 1.5)	1.3 (1.1, 1.5)	1.4 (1.2, 1.8)	<0.001
Lactate (mmol/L)	1.9 (1.4, 2.6)	1.8 (1.3, 2.5)	2.0 (1.4, 2.9)	<0.001
SpO_2_ (%)	97.3 (95.8, 98.6)	97.3 (95.9, 98.6)	97.0 (95.4, 98.5)	<0.001
PH	7.4 (7.3, 7.4)	7.4 (7.3, 7.4)	7.4 (7.3, 7.4)	<0.001
Bicarbonate (mmHg)	23.0 (20.3, 25.3)	23.0 (20.6, 25.3)	22.3 (19.0, 25.3)	<0.001
Glucose (mg/dl)	130.2 (109.0, 163.0)	128.5 (109.0, 159.3)	136.5 (110.8, 175.5)	<0.001
Comorbidities, n (%)				
Pneumonia	4,385 (24.1)	2,909 (21.4)	1,476 (32.3)	<0.001
COPD	2,302 (12.6)	1,634 (12.0)	668 (14.6)	<0.001
Hypertension	12,075 (66.4)	8,989 (66.0)	3,086 (67.5)	0.069
CHF	6,125 (33.7)	4,222 (31.0)	1,903 (41.6)	<0.001
MI	3,294 (18.1)	2,358 (17.3)	936 (20.5)	<0.001
Renal disease	4,495 (24.7)	3,063 (22.5)	1,432 (31.3)	<0.001
Liver disease	2,036 (11.2)	1,402 (10.3)	634 (13.9)	<0.001
Osteoporosis	908 (5.0)	674 (4.9)	234 (5.1)	0.653
Diabetes	5,904 (32.4)	4,398 (32.3)	1,506 (32.9)	0.428
Cancer	2,427 (13.3)	1,425 (10.5)	1,002 (21.9)	<0.001
Organ support, n (%)				
Mechanical ventilation	9,977 (54.8)	7,352 (54.0)	2,625 (57.4)	<0.001
Vasopressors	9,218 (50.7)	6,666 (48.9)	2,552 (55.8)	<0.001
RRT	1,399 (7.7)	880 (6.5)	519 (11.3)	<0.001
ECMO	22 (0.1)	11 (0.1)	11 (0.2)	0.007
Severity score				
SOFA	4.1 (2.7, 6.2)	4.0 (2.6, 5.9)	4.8 (3.0, 7.0)	<0.001
SAPS II	38.0 (31.0, 47.0)	36.0 (29.0, 44.0)	45.0 (37.0, 54.0)	<0.001
CCI	6.0 (4.0, 8.0)	5.0 (4.0, 7.0)	7.0 (5.0, 9.0)	<0.001
Outcomes				
Hospital LOS days	8.3 (5.3, 14.2)	8.5 (5.5, 14.5)	7.9 (4.3, 13.8)	<0.001
ICU LOS days	3.1 (1.9, 6.2)	3.0 (1.8, 5.8)	3.9 (2.2, 7.5)	<0.001

BMI, body mass index; WBC, white blood cell; BUN, blood urea nitrogen; PT, prothrombin time; APTT, activated partial thromboplastin time; INR, international standard ratio; SpO_2_, pulse oxygen saturation; COPD, chronic obstructive pulmonary disease; CHF, congestive heart failure; MI, myocardial infarction; RRT, renal replacement therapy; ECMO, extracorporeal membrane oxygenation; SOFA, sequential organ failure assessment; SAPA II, simplified acute physiology score; CCI, Charlson comorbidities index; Hospital LOS days, hospital length of stay days; ICU LOS days, intensive care unit length of stay days.

### 28-day and 90-day stepwise Cox proportional hazards regression analysis

The covariates with statistical significance (*P* < 0.05) in Tables 3, 4 were subjected to univariate Cox regression analysis and were subsequently incorporated into a multivariate stepwise Cox proportional hazards regression analysis framework. The findings indicated a significant association between the prophylactic administration of PPIs and all-cause mortality within 90 days of admission to ICU (HR, 0.74; 95% CI, 0.64–0.82; *P* < 0.001) (Table 6). Conversely, no statistically significant relationship was identified between the prophylactic use of PPI and all-cause mortality within 28 days of ICU admission (*P* = 0.910). In the 28-day stepwise Cox proportional hazards regression model, all-cause mortality was correlated with various factors, including age, BMI, pantoprazole, SOFA, CCI, shock, AKI, MV, lactate, APTT, INR, and lymphocytes. Similarly, the 90-day stepwise Cox proportional hazards regression model identified predictors of all-cause mortality within 90 days of ICU admission, including age, BMI, ICU LOS days, PPI, lansoprazole, pantoprazole, SOFA, CCI, shock, AKI, vasopressors, MV, ECMO, RRT, lactate, APTT, INR, lymphocytes, platelets, and creatinine.

Univariate ROC curve analysis was performed on the covariates related to all-cause death in Tables 5, 6. The values of AUC, sensitivity, and specificity are shown in Tables 7, 8. The analysis showed that age, lymphocyte (%), and related severity scores (SOFA and CCI) showed significant AUC values, significantly affecting the performance of the model. It is worth noting that in the 28-day model, the AUC values of pantoprazole and APTT were lower; in the 90-day model, the AUC values of vasoactive drugs, ECMO, RRT, and platelets were lower. Therefore, we considered removing the above variables with lower AUC from the model. In addition, it was found for the 90-day model that the prophylactic use of PPI in patients with sepsis was statistically significant, with an AUC of 0.52 (95% CI: 0.51–0.53); the AUC values of lansoprazole and pantoprazole were 0.52 (0.51, 0.52) and 0.52 (0.51, 0.53), respectively.

**TABLE 5:** Stepwise Cox proportional hazard regression analysis of the 28-day mortality rate.

Variables	Univariate			Multivariate		
	HR	HR (95% CI)	*P*	HR	HR (95% CI)	*P*
Age (yr)	1.03	(1.02, 1.03)	<0.001	1.01	(1.01, 1.02)	<0.001
Gender						
Female		Reference				
Male	0.96	(0.89, 1.04)	0.052			
BMI (kg/m^2^)	0.97	(0.97, 0.98)	<0.001	0.97	(0.96, 0.98)	<0.001
ICU LOS days	1.01	(1.00, 1.01)	0.087			
PPI						
0		Reference				
1	1.00	(0.92, 1.08)	0.910			
Lansoprazole						
0		Reference				
2	1.10	(0.97, 1.25)	0.148			
3	0.46	(0.11, 1.83)	0.268			
4	0.00	(0.00, 4.86 × 10^100^)	0.970			
Pantoprazole						
0		Reference			Reference	
1	2.24	(2.02, 2.48)	<0.001	1.77	(1.59, 1.96)	<0.001
2	0.84	(0.73, 0.97)	0.014	0.80	(0.69, 0.92)	0.002
3	0.47	(0.07, 3.37)	0.455	0.42	(0.06, 2.98)	0.384
4	0.77	(0.67, 0.88)	<0.001	0.63	(0.55, 0.73)	<0.001
SOFA	1.14	(1.13, 1.16)	<0.001	1.04	(1.03, 1.06)	<0.001
CCI	1.17	(1.15, 1.19)	<0.001	1.13	(1.12, 1.15)	<0.001
Shock						
0		Reference			Reference	
1	2.47	(2.26, 2.70)	<0.001	1.60	(1.45, 1.76)	<0.001
AKI						
0		Reference			Reference	
1	2.59	(2.28, 2.93)	<0.001	1.87	(1.64, 2.13)	<0.001
Vasopressors						
0		Reference				
1	1.59	(1.46, 1.73)	<0.001			
MV						
<48 h		Reference			Reference	
≥48 h	2.15	(1.97, 2.34)	<0.001	1.64	(1.49, 1.80)	<0.001
ECMO						
0		Reference				
1	2.68	(1.28, 5.63)	0.009			
RRT						
0		Reference				
1	1.82	(1.60, 2.06)	<0.001			
Lactate (mmol/L)	1.21	(1.19, 1.23)	<0.001	1.15	(1.13, 1.17)	<0.001
APTT (s)	1.01	(1.01, 1.02)	<0.001	1.00	(1.00, 1.01)	<0.001
INR	1.22	(1.19, 1.25)	<0.001	1.08	(1.05, 1.12)	<0.001
Lymphocytes (%)	0.97	(0.96, 0.97)	<0.001	0.98	(0.98, 0.99)	<0.001
Platelet (g/dL)	0.07	(1.00, 1.00)	0.071			
Creatinine (mg/dL)	1.07	(1.05, 1.10)	<0.001			

HR, hazard ratio; CI, confidence interval; BMI, body mass index; ICU LOS days, intensive care unit length of stay days; PPI, proton pump inhibitors; SOFA, sequential organ failure assessment; CCI, Charlson comorbidities index; AKI, acute kidney injury; MV, mechanical ventilation; ECMO, extracorporeal membrane oxygenation; CRRT, continuous renal replacement therapy; APTT, activated partial thromboplastin time; INR, international standard ratio.

**TABLE 6:** Stepwise Cox proportional hazards regression analysis of the 90-day mortality rate.

Variables	Univariate			Multivariate		
	HR	HR (95% CI)	*P*	HR	HR (95% CI)	*P*
Age (yr)	1.03	(1.03, 1.03)	<0.001	1.02	(1.01, 1.02)	<0.001
Gender						
Female		Reference				
Male	0.94	(0.88, 1.01)	0.091			
BMI (kg/m^2^)	0.97	(0.97, 0.98)	<0.001	0.97	(0.96, 0.97)	<0.001
ICU LOS days	1.02	(1.01, 1.02)	<0.001	0.98	(0.97, 0.99)	<0.001
PPI						
0		Reference			Reference	
1	1.14	(1.06, 1.22)	<0.001	0.74	(0.64, 0.86)	<0.001
Lansoprazole						
0		Reference			Reference	
2	1.32	(1.19, 1.46)	<0.001	1.32	(1.16, 1.50)	<0.001
3	0.66	(0.25, 1.77)	0.414	0.85	(0.26, 2.78)	0.785
4	0.45	(0.06, 3.21)	0.427	0.68	(0.10, 4.83)	0.698
Pantoprazole						
0		Reference			Reference	
1	2.10	(1.92, 2.30)	<0.001	2.38	(2.03, 2.79)	<0.001
2	1.02	(0.91, 1.14)	0.778	1.09	(0.91, 1.30)	0.376
3	0.70	(0.17, 2.79)	0.611	0.94	(0.18, 5.05)	0.946
4	0.99	(0.89, 1.10)	0.872	1.04	(0.88, 1.24)	0.626
SOFA	1.11	(1.10, 1.13)	<0.001	1.06	(1.04, 1.07)	<0.001
CCI	1.20	(1.18, 1.21)	<0.001	1.17	(1.15, 1.18)	<0.001
Shock						
0		Reference			Reference	
1	2.27	(2.10, 2.45)	<0.001	1.63	(1.49, 1.77)	<0.001
AKI						
0		Reference			Reference	
1	1.99	(1.81, 2.19)	<0.001	1.55	(1.40, 1.72)	<0.001
Vasopressors						
0		Reference				
1	1.34	(1.25, 1.44)	<0.001	0.88	(0.80, 0.96)	0.003
MV						
<48 h		Reference			Reference	
≥48 h	1.92	(1.78, 2.07)	<0.001	1.81	(1.63, 2.00)	<0.001
ECMO						
0		Reference				
1	2.65	(1.38, 5.10)	0.003	2.01	(1.03, 3.91)	0.040
RRT						
0		Reference			Reference	
1	1.69	(1.51, 1.89)	<0.001	1.23	(1.07, 1.42)	0.003
Lactate (mmol/L)	1.17	(1.16, 1.19)	<0.001	1.12	(1.10, 1.14)	<0.001
APTT (s)	1.01	(1.01, 1.01)	<0.001	1.01	(1.01, 1.01)	0.036
INR	1.21	(1.18, 1.24)	<0.001	1.09	(1.06, 1.13)	<0.001
Lymphocytes (%)	0.97	(0.96, 0.97)	<0.001	0.98	(0.98, 0.99)	<0.001
Platelet (g/dL)	1.01	(1.01, 1.01)	<0.001	1.01	(1.01, 1.01)	<0.001
Creatinine (mg/dL)	1.07	(1.05, 1.08)	<0.001	0.95	(0.92, 0.98)	<0.001

HR, hazard ratio; CI, confidence interval; BMI, body mass index; ICU LOS days, intensive care unit length of stay days; PPI, proton pump inhibitors; SOFA, sequential organ failure assessment; CCI, Charlson comorbidities index; AKI, acute kidney injury; MV, mechanical ventilation; ECMO, extracorporeal membrane oxygenation; CRRT, continuous renal replacement therapy; APTT, activated partial thromboplastin time; INR, international standard ratio.

**TABLE 7:** 28-day single factor ROC curve.

Variables	AUC (95% CI)	Accuracy (95% CI)	Sensitivity (95% CI)	Specificity (95% CI)	Cut off
Age	0.62 (0.61, 0.63)	0.65 (0.64, 0.65)	0.68 (0.67, 0.69)	0.51 (0.50, 0.53)	74.921
BMI (kg/m^2^)	0.57 (0.56, 0.58)	0.43 (0.42, 0.44)	0.42 (0.41, 0.43)	0.48 (0.46, 0.50)	26.367
Pantoprazole	0.50 (0.49, 0.51)	0.57 (0.57, 0.58)	0.60 (0.59, 0.61)	0.45 (0.43, 0.46)	-
SOFA	0.60 (0.59, 0.61)	0.63 (0.62, 0.64)	0.66 (0.65, 0.67)	0.50 (0.48, 0.51)	5.021
CCI	0.65 (0.64, 0.66)	0.62 (0.61, 0.63)	0.63 (0.62, 0.64)	0.58 (0.57, 0.60)	6.5
Shock	0.59 (0.58, 0.60)	0.76 (0.75, 0.76)	0.85 (0.85, 0.86)	0.32 (0.30, 0.34)	-
AKI	0.58 (0.57, 0.58)	0.39 (0.38, 0.39)	0.28 (0.27, 0.29)	0.88 (0.87, 0.89)	-
MV > 48 h	0.59 (0.58, 0.59)	0.73 (0.73, 0.74)	0.81 (0.81, 0.82)	0.36 (0.34, 0.37)	-
Lactate (mmol/L)	0.59 (0.58, 0.60)	0.68 (0.68, 0.69)	0.75 (0.74, 0.75)	0.39 (0.37, 0.41)	2.5
APTT (s)	0.56 (0.55, 0.57)	0.69 (0.68, 0.69)	0.76 (0.75, 0.77)	0.35 (0.34, 0.37)	38.026
INR	0.60 (0.58, 0.61)	0.71 (0.71, 0.72)	0.79 (0.78, 0.79)	0.38 (0.37, 0.40)	1.537
Lymphocytes (%)	0.61 (0.60, 0.62)	0.44 (0.44, 0.45)	0.46 (0.45, 0.47)	0.36 (0.35, 0.38)	11.5

BMI, body mass index; ICU LOS days, intensive care unit length of stay days; SOFA, sequential organ failure assessment; CCI, Charlson comorbidities index; AKI, acute kidney injury; MV, mechanical ventilation; INR, international standard ratio.

**TABLE 8:** 90-day single factor ROC curve.

Variables	AUC (95% CI)	Accuracy (95% CI)	Sensitivity (95% CI)	Specificity (95% CI)	Cut off
Age	0.63 (0.62, 0.64)	0.65 (0.64, 0.65)	0.69 (0.69, 0.70)	0.51 (0.49, 0.52)	74.985
BMI (kg/m^2^)	0.58 (0.57, 0.59)	0.42 (0.42, 0.43)	0.41 (0.40, 0.41)	0.47 (0.46, 0.49)	26.376
ICU LOS days	0.58 (0.57, 0.59)	0.58 (0.57, 0.58)	0.59 (0.58, 0.60)	0.54 (0.53, 0.55)	3.655
PPI	0.52 (0.51, 0.53)	0.51 (0.51, 0.52)	0.51 (0.50, 0.52)	0.53 (0.51, 0.54)	-
Lansoprazole	0.52 (0.51, 0.52)	0.71 (0.70, 0.71)	0.90 (0.89, 0.90)	0.13 (0.12, 0.14)	-
Pantoprazole	0.52 (0.51, 0.53)	0.57 (0.57, 0.58)	0.61 (0.60, 0.62)	0.46 (0.45, 0.48)	-
SOFA	0.58 (0.57, 0.59)	0.60 (0.60, 0.61)	0.64 (0.64, 0.65)	0.48 (0.46, 0.49)	4.957
CCI	0.68 (0.67, 0.69)	0.57 (0.56, 0.58)	0.51 (0.50, 0.52)	0.75 (0.73, 0.76)	5.5
Shock	0.58 (0.57, 0.58)	0.72 (0.71, 0.72)	0.86 (0.86, 0.87)	0.86 (0.86, 0.87)	-
AKI	0.56 (0.56, 0.57)	0.42 (0.42, 0.43)	0.28 (0.27, 0.29)	0.84 (0.83, 0.86)	-
Vasopressors	0.53 (0.53, 0.54)	0.52 (0.52, 0.53)	0.51 (0.50, 0.52)	0.56 (0.54, 0.57)	-
MV > 48 h	0.57 (0.56, 0.58)	0.69 (0.69, 0.70)	0.82 (0.81, 0.83)	0.32 (0.31, 0.33)	-
ECMO	0.50 (0.50, 0.50)	0.75 (0.74, 0.75)	1.00 (1.00, 1.00)	0.00 (0.00, 0.00)	-
RRT	0.52 (0.52, 0.53)	0.73 (0.72, 0.74)	0.94 (0.93, 0.94)	0.11 (0.10, 0.12)	-
Lactate (mmol/L)	0.56 (0.55, 0.57)	0.67 (0.66, 0.68)	0.79 (0.78, 0.80)	0.32 (0.30, 0.33)	2.646
APTT (s)	0.56 (0.55, 0.57)	0.64 (0.63, 0.64)	0.72 (0.72, 0.73)	0.38 (0.36, 0.39)	36.7
INR	0.60 (0.59, 0.61)	0.69 (0.68, 0.70)	0.80 (0.79, 0.80)	0.37 (0.36, 0.38)	1.537
Lymphocytes (%)	0.61 (0.60, 0.62)	0.43 (0.42, 0.44)	0.45 (0.44, 0.46)	0.38 (0.36, 0.39)	11.5
Platelet (g/dL)	0.51 (0.50, 0.52)	0.63 (0.62, 0.64)	0.73 (0.72, 0.74)	0.33 (0.31, 0.34)	242.417
Creatinine (mg/dL)	0.59 (0.58, 0.60)	0.60 (0.59, 0.60)	0.61 (0.60, 0.62)	0.55 (0.54, 0.56)	1.154

BMI, body mass index; ICU LOS days, intensive care unit length of stay days; PPI, proton pump inhibitors; SOFA, sequential organ failure assessment; CCI, Charlson comorbidities index; AKI, acute kidney injury; MV, mechanical ventilation; INR, international standard ratio.

**FIGURE 3:**
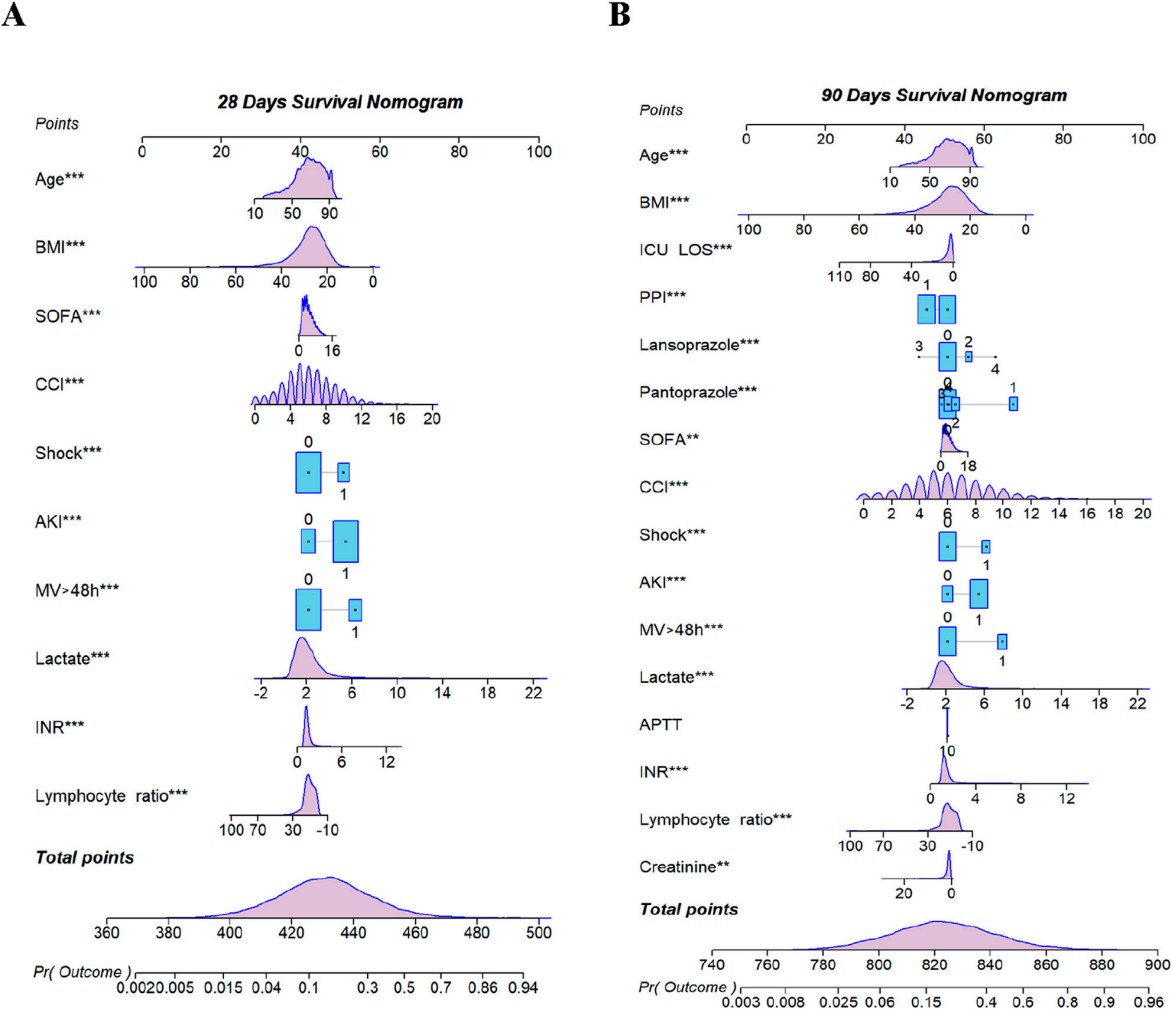
Nomogram for predicting mortality. **(A)** 28-day Model. **(B)** 90-day Model. BMI, body mass index; ICU Los, intensive care unit length of stay; PPI, proton pump inhibitors; SOFA, sequential organ failure assessment; CCI, Charlson comorbidities index; AKI, acute kidney injury; MV, mechanical ventilation; APTT, activated partial thromboplastin time; INR, international normalized ratio. ** < 0.01; *** < 0.001.

Based on stepwise Cox proportional hazard regression analysis combined with single factor ROC curve and clinical decision evaluation, this study constructed predictive models for 28- and 90-day prognoses, respectively. The 28-day prognostic model included ten key variables, and the 90-day prognostic model included 16. In order to visually present the model structure, the variable weights and prediction probabilities of the two models are further visually displayed through the nomogram (Figure 3).

**FIGURE 4:**
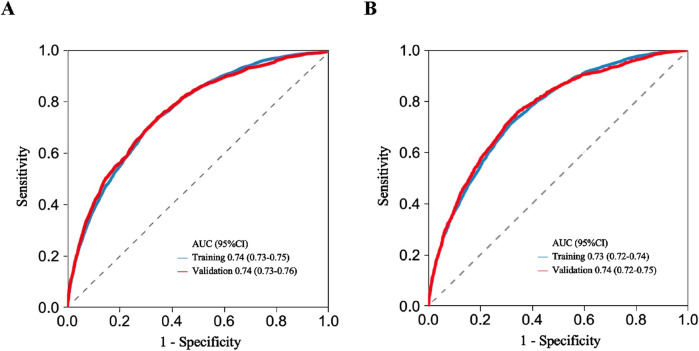
ROC curve analysis used for discriminating model performance. **(A)** 28-day model ROC curve. **(B)** 90-day model ROC curve. AUC, area under curve; CI, confidence interval.

**FIGURE 5:**
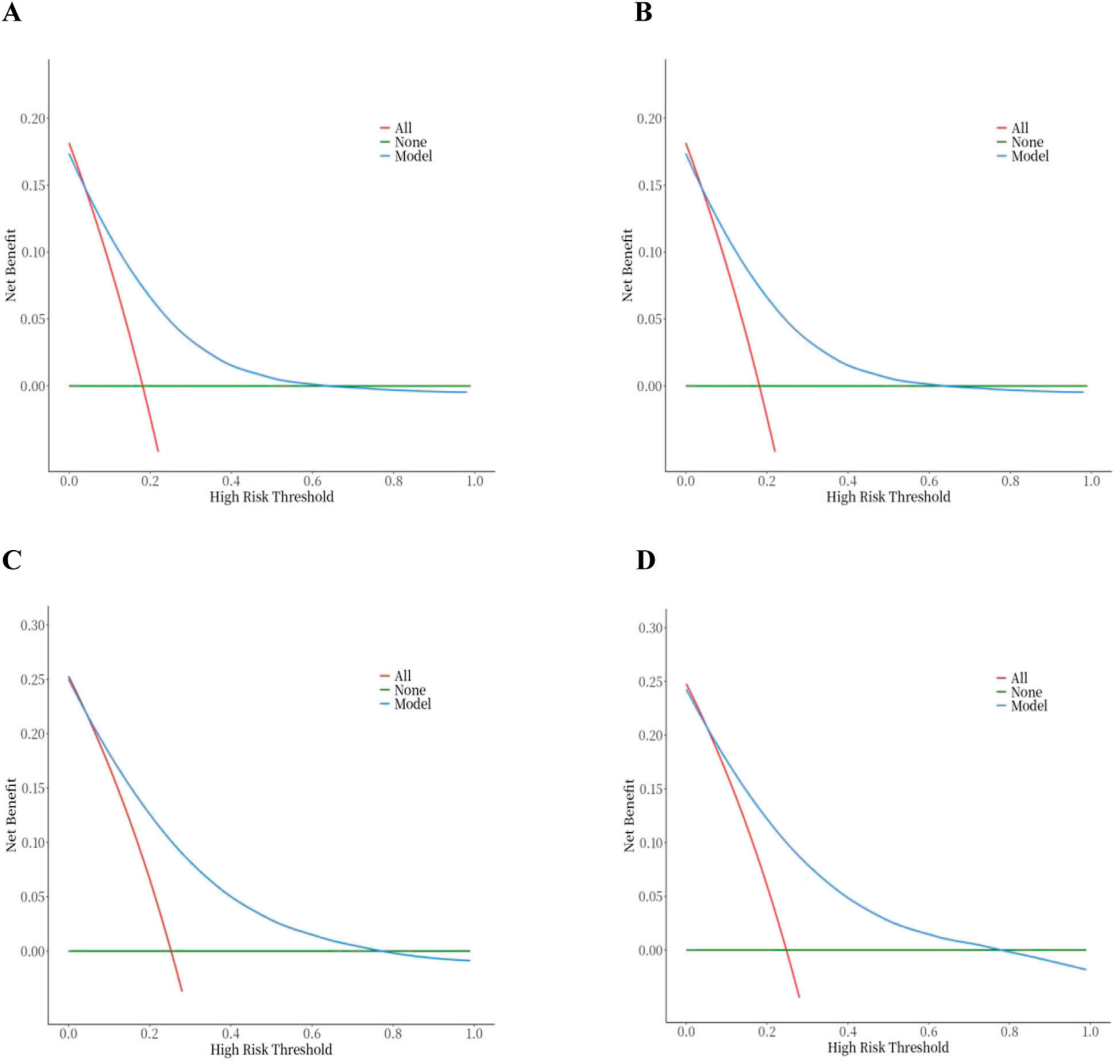
Decision curve analysis of the nomogram. **(A)** 28‐day model in the training set. **(B)** 28‐day model in the testing set. **(C)** 90‐day model in the training set. **(D)** 90‐day model in the testing set.

**FIGURE 6:**
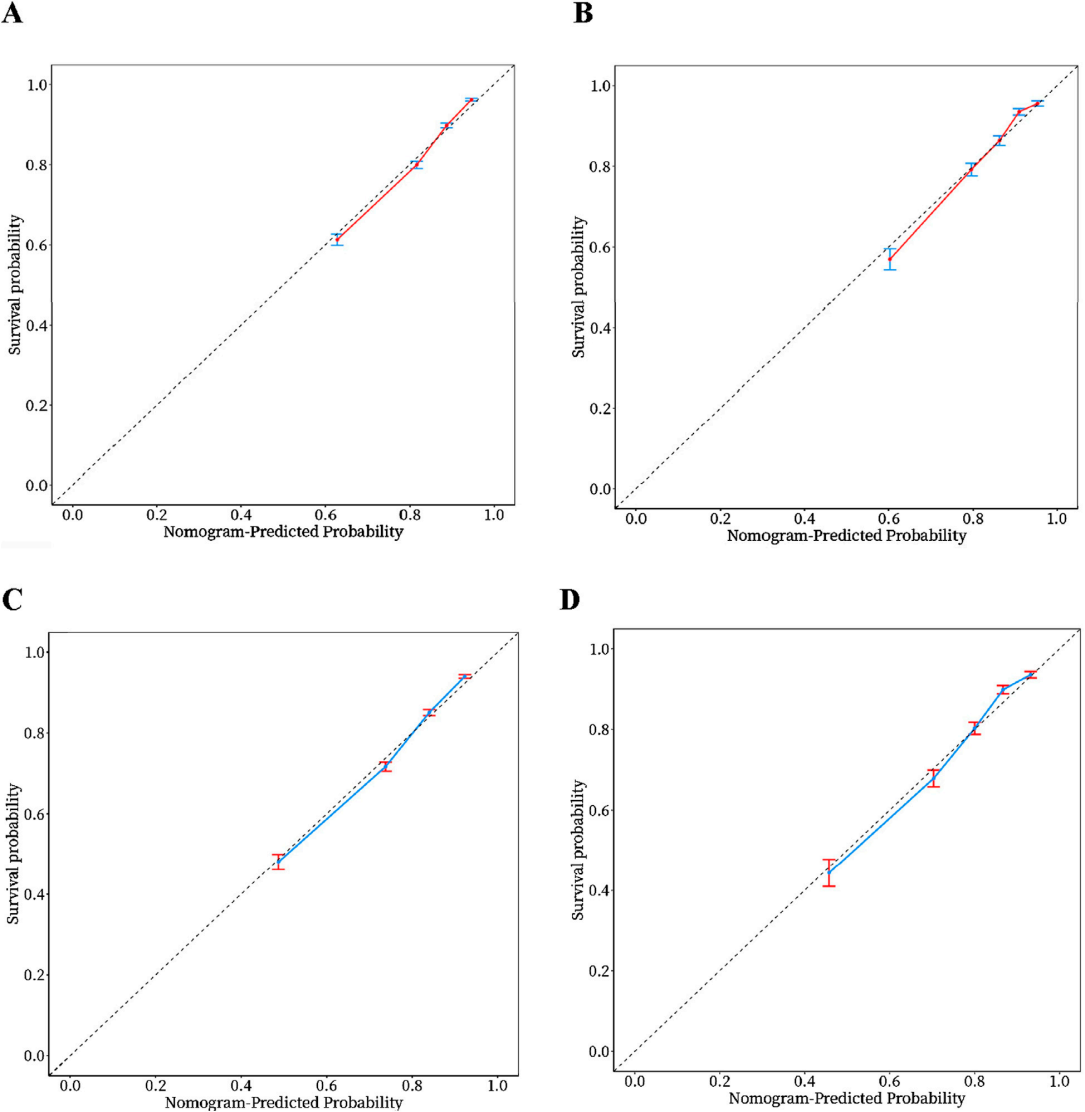
Calibration curves of the nomogram. **(A)** 28‐day model in the training set. **(B)** 28‐day model in the validation set. **(C)** 90‐day model in the training set. **(D)** 90‐day model in the validation set.

In order to evaluate the predictive performance of the model, ROC curve, calibration curve, and decision curve (DCA) were used for comprehensive verification. First, 18,198 patients were randomly divided into a training set (12,739 cases) and a validation set (5,459 cases) at a respective 7:3 ratio. The results showed that in the 28-day prognostic model, the AUCs of the training and validation sets were 0.74 (95% CI 0.73–0.75) and 0.74 (95% CI 0.73–0.76), respectively. In the 90-day prognostic model, the AUCs were 0.73 (95% CI 0.72–0.74) and 0.74 (95% CI 0.72–0.75), respectively. Further comparison with traditional indicators found that the prediction accuracy of the 28- and 90-day models was significantly better than that of CCI (AUC 65% and 68%) and SOFA (AUC 60% and 58%), indicating that the new model has more advantages in prognostic evaluation (Figure 4). In order to evaluate the clinical applicability of the model, the net benefit was further quantified by decision DCA. As shown in Figure 5, within the threshold probability range of 0.1–0.6, the model can significantly improve the net benefit compared with the extreme strategy of “treating all patients” or “not treating any patients”; this suggests that it has practical application value in this interval. However, when the threshold probability exceeds 0.6, the confidence interval between the net benefit of the model and the “no treatment” strategy overlaps, indicating that its clinical significance under the high-risk threshold is limited. Based on the results of calibration (Figure 6), discrimination ability (AUC > 0.7), and DCA, the nomogram can provide a reliable tool for individualized prognosis evaluation of patients with sepsis.

### The primary outcome and secondary outcomes

**FIGURE 7:**
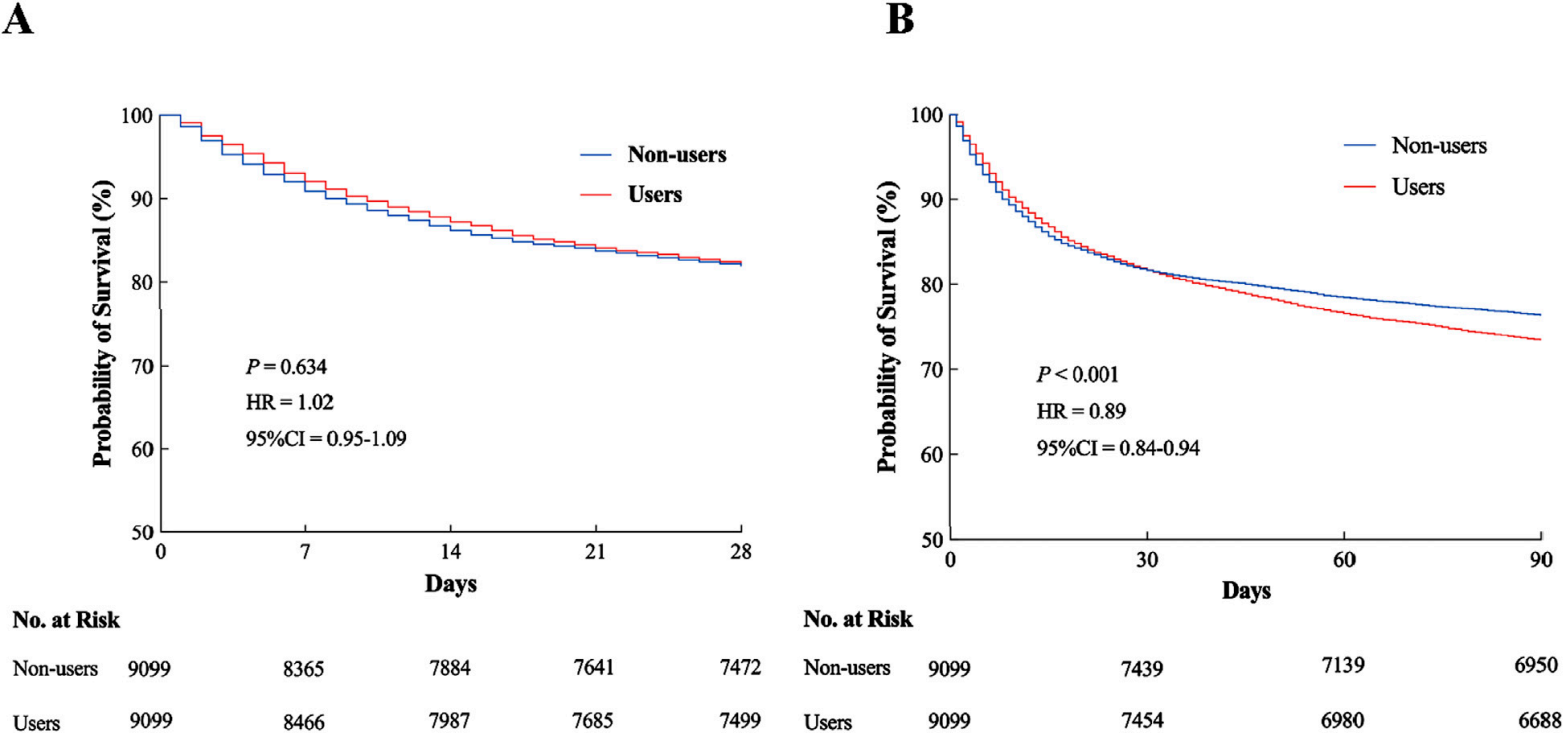
Kaplan–Meier survival analysis curves. **(A)** All-cause mortality rate at 28 days in ICU. **(B)** All-cause mortality rate at 90 days in ICU. HR, hazard ratio; CI, confidence interval; ICU, intensive care unit.

The overall mortality rate increased from 18% at 28 days to 25% at 90 days post ICU admission. Despite this temporal trend, a statistically significant difference in all-cause mortality emerged between the PPI Users and Non-users groups, specifically at 90 days (*P* < 0.001; Figure 7B). Although the survival probability of PPI Users began to decline relative to Non-users as early as 28 days (HR = 1.02, 95% CI = 0.95−1.09, *P* = 0.634; Figure 7A), this short-term divergence did not reach statistical significance. However, during the 90-day follow-up, the survival disparity between the groups widened substantially, with PPI Users exhibiting a 7% absolute increase in mortality compared to Non-users. These findings imply that prophylactic PPI use might be associated with diminished long-term survival, warranting further investigation into its potential adverse effects.

As shown in Tables 2, 3, the Users group exhibited significantly prolonged hospital stays compared to the Non-users, while their ICU stays were notably shorter. Specifically, the median hospital stay duration was 8.7 days (IQR 5.6–15.0) in the Users group versus 6.9 days (IQR 3.7–11.9) in the Non-users group (*P* < 0.001) at 28 days, with a similar trend observed at 90 days (8.5 vs. 7.9 days; *P* = 0.012). Conversely, the median ICU stay was reduced in the Users group (3.0 days, IQR 1.8–6.0) relative to the Non-users (4.1 days, IQR 2.2–7.4) at 28 days (*P* = 0.003), and this reduction persisted at 90 days (3.0 vs. 3.9 days; *P* = 0.008).

### Adverse reactions

**FIGURE 8:**
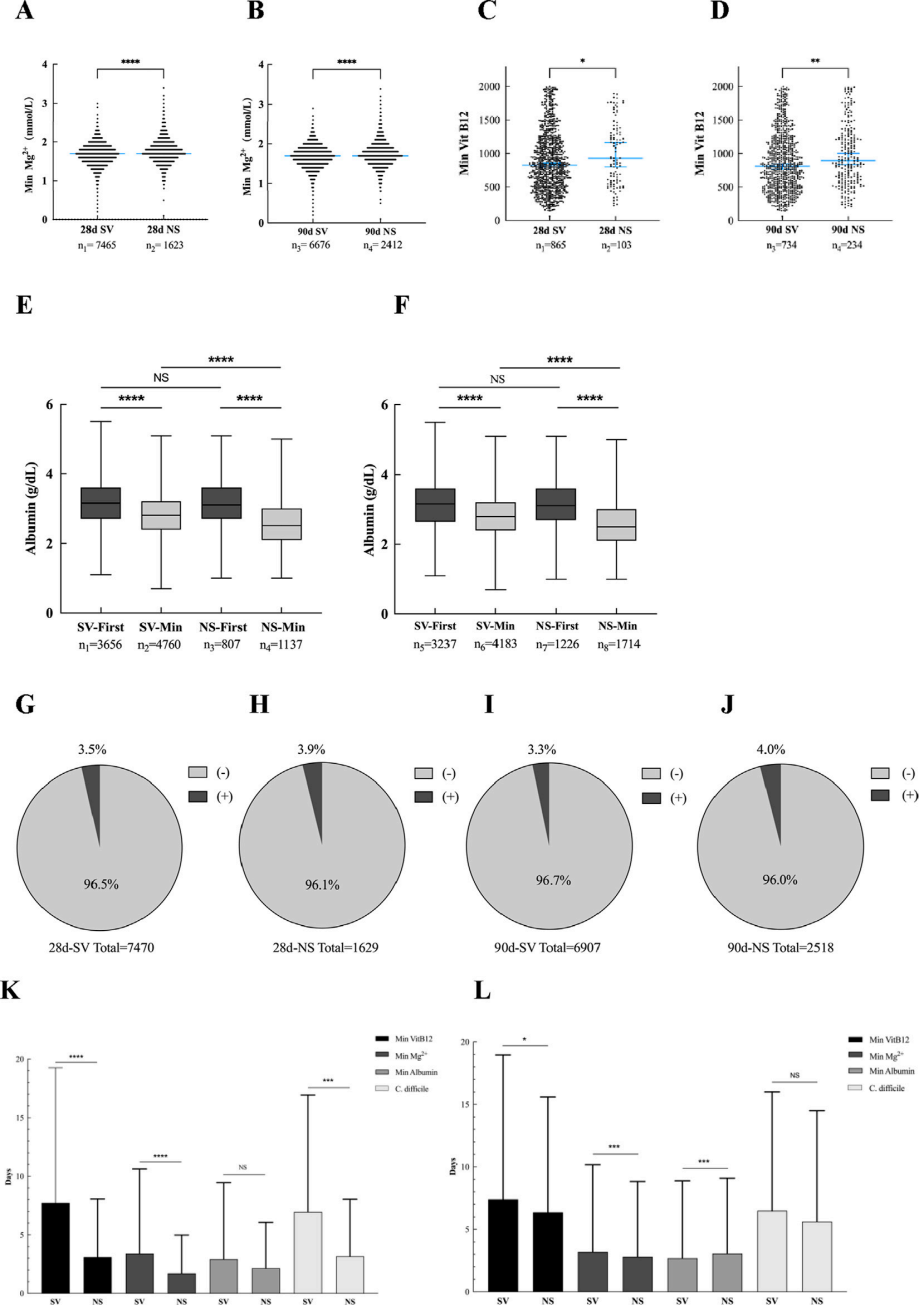
Adverse reactions related to PPI use. **(A)** Mg^2+^ lowest value (28-day post-ICU). **(B)** Mg^2+^ lowest value (90-day post-ICU). **(C)** Vit B12 lowest value (28-day post-ICU). **(D)** Vit B12 lowest value (90-day post-ICU). **(E)** Initial and minimum values of albumin (28-day post-ICU). **(F)** Initial and minimum values of albumin (90-day post-ICU). **(G)** Proportion of positive fecal *C. difficile* (28-day Survival group). **(H)** Proportion of positive fecal *C. difficile* (28-day Non-survival group). **(I)** The proportion of positive fecal *C. difficile* (90-day Survival group). **(J)** Proportion of positive fecal *C. difficile* (90-day Non-survival group). **(K)** Number of days with lowest value or positive rate (28-day Post-ICU). **(L)** Number of days with lowest value or positive rate (90-day Post-ICU). * < 0.05; *** < 0.001; **** < 0.0001.

This study systematically evaluated the potential adverse effects of prophylactic PPI use, including electrolyte disorders (hypomagnesemia), nutritional deficiencies (vitamin B12), hypoproteinemia, and opportunistic infections. As shown in Figures 8A, B, the median serum magnesium level was 1.7 mmol/L, and no hypomagnesemia occurred in any patients. In Figures 8C, D, the median of vitamin B12 was 825 and 810 pg/mL in the Survival group and 928 and 897 pg/mL in the Non-survival group, respectively. No vitamin B12 deficiency was observed. In terms of hypoproteinemia, the median baseline albumin at admission in the Survival and Non-survival groups was 3.15 and 3.10 g/dL, respectively. However, within 2 ‐3 days after admission to ICU (Figures 8E,F), the albumin in the Survival group decreased to a minimum of 2.8 g/dL (Figure 8K), while the albumin in the Non-survival group further decreased to 2.5 g/dL (Figure 8L), suggesting that the early nutritional risk of ICU increased. Opportunistic infection analysis showed that the average incidence of infection in the Survival group was 3.4% (Figure 8G), which occurred on the seventh day of ICU admission. The average incidence of infection in the Non-survival group was significantly increased to 3.95% (Figure 8I), and the infection time was earlier (average 4 days). It is worth noting that the proportion of *C. difficile* infection in the Non-survival group was higher, suggesting that it was closely related to poor prognosis.

### Types and administration methods of PPI

**FIGURE 9:**
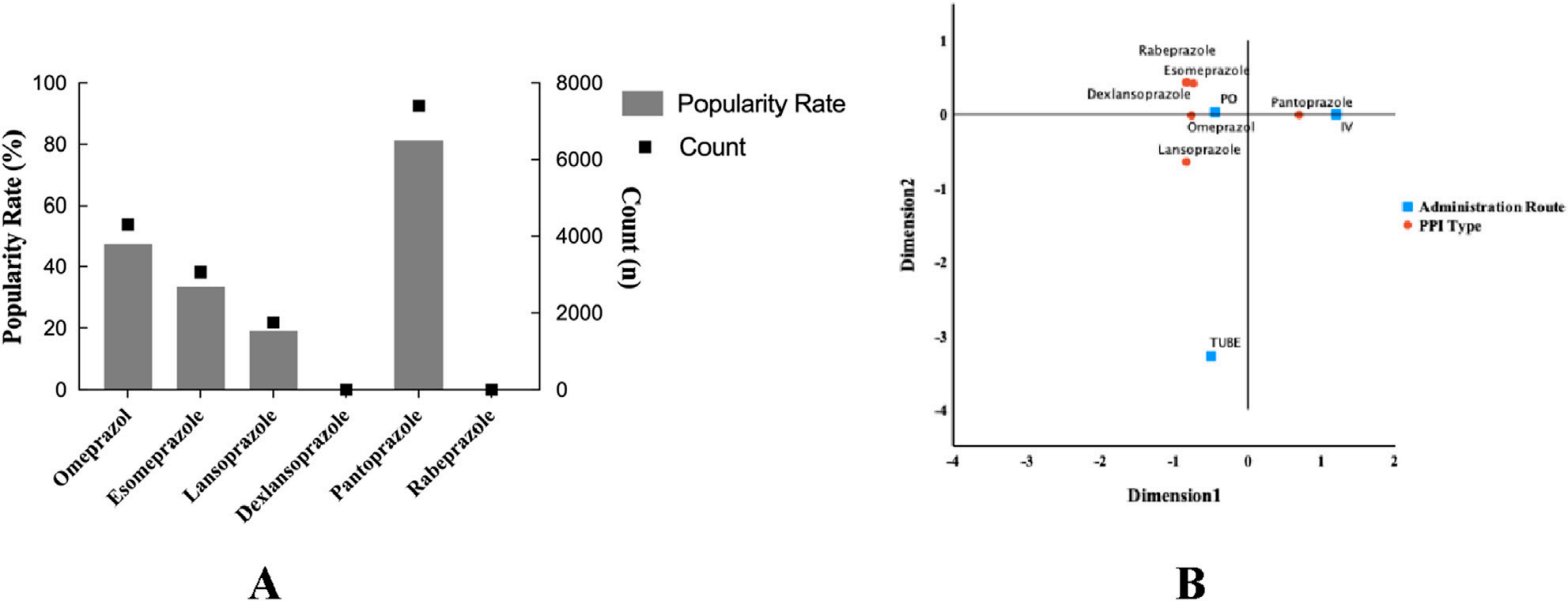
Column dotted line chart. Types and administration of PPI for prophylactic use in sepsis patients. **(A)** Different types and ratios of PPI. **(B)** Corresponding analysis chart; different administration methods of proton pump inhibitors. PPI, proton pump inhibitors; PO, per os; IV, intravenous injection or other intravenous routes of administration; TUBE, gastrostomy tube.

The distribution of PPI use was significantly different in ICU adult patients with sepsis. Specifically, the use of pantoprazole accounted for the highest proportion (81.3%, 7,395), while the use of dexlansoprazole was the lowest (3, 0.03%). Among the remaining PPI varieties, the use frequency of omeprazole, esomeprazole, and lansoprazole decreased in turn. From the analysis of the route of administration, oral administration is dominant, while enteral routes such as gastrostomy tube are less used. It is worth noting that the administration of pantoprazole is mainly intravenous infusion, followed by oral administration (Figure 9).

### Subgroup analysis

In order to further explore the potential confounding effects of prophylactic PPI use on the prognosis of patients with sepsis, subgroup interaction analysis was performed by multivariate regression model. The results showed that there was a significant interaction between disease severity (SOFA score) subgroup and PPI use (interaction *P* = 0.021) in the 28-day all-cause mortality risk, suggesting that the effect of PPI on short-term prognosis may change dynamically with the severity of the disease. At the same time, the mechanical ventilation subgroup showed a stronger interaction (interaction *P* < 0.001), indicating that longer mechanical ventilation may increase the short-term risk of PPI. Further analysis of the 90-day all-cause mortality risk found that the interaction effect of the mechanical ventilation subgroup continued to be significant (interaction *P* < 0.001), while the blood lactic acid subgroup also showed a significant interaction (interaction *P* = 0.020), suggesting that lactic acid metabolism disorder may mediate the long-term poor prognosis of PPI (Table 9). It is worth noting that the moderating effect of the SOFA score was not significant during the 90-day follow-up (interaction *P* = 0.181), highlighting the heterogeneity of mechanisms in different time windows. In summary, the severity of disease, mechanical ventilation status, and lactic acid metabolism disorder are the key regulatory factors associated with PPI and prognosis (Table 10). The interaction effect shows time-series dynamic characteristics at 28 and 90 days, which provide a risk stratification basis for individualized PPI medication.

**TABLE 9:** Subgroup analysis of the association between PPI and 28-day all-cause mortality risk.

Variables	n (%)	HR (95% CI)		P	P for interaction
All patients	18,198 (100.0)	0.98 (0.91, 1.05)	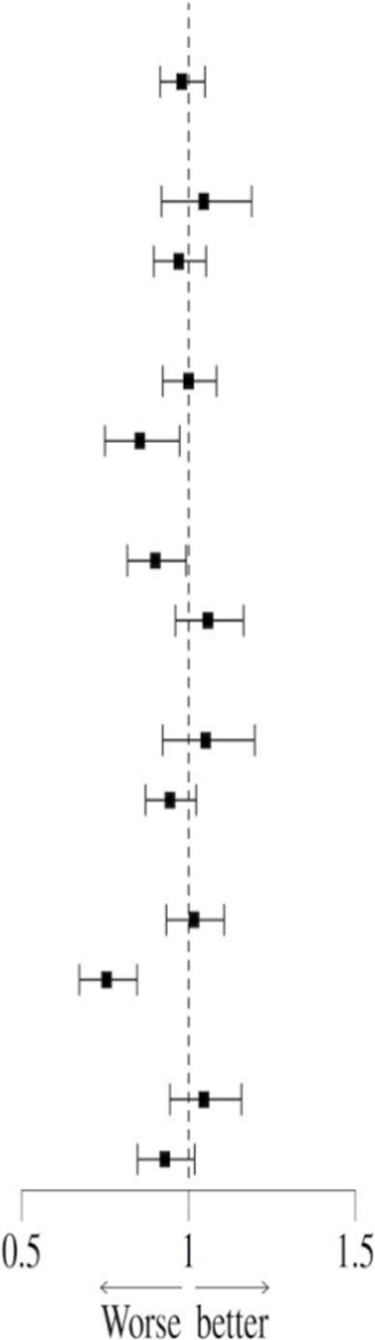	0.551	
Age (yr)					0.342
<65	7,319 (40.2)	1.05 (0.92, 1.19)		0.501	
≥65	10,879 (59.8)	0.97 (0.90, 1.05)		0.470	
ICU LOS days					0.057
<7	14,208 (78.1)	1.00 (0.92, 1.08)		0.999	
≥7	3,990 (21.9)	0.85 (0.75, 0.97)		0.018	
SOFA					0.021
<5	11,277 (62.0)	0.90 (0.82, 0.99)		0.035	
≥5	6,921 (38.0)	1.06 (0.96, 1.16)		0.253	
CCI					0.171
<6	8,148 (44.8)	1.05 (0.92, 1.20)		0.453	
≥6	10,050 (55.2)	0.94 (0.87, 1.02)		0.160	
MV					<.001
<48 h	14,264 (78.4)	1.02 (0.93, 1.11)		0.712	
≥48 h	3,934 (21.6)	0.75 (0.67, 0.85)		<.001	
Lactate (mmol/L)					0.089
<2	9,913 (54.5)	1.05 (0.94, 1.16)		0.391	
≥2	8,285 (45.5)	0.93 (0.85, 1.02)		0.115	
				

ICU LOS days, intensive care unit length of stay days; SOFA, sequential organ failure assessment; CCI, Charlson comorbidities index; MV, mechanical ventilation.

**TABLE 10:** Subgroup analysis of the association between PPI and 90-day all-cause mortality risk.

Variables	n (%)	HR (95% CI)		P	P for interaction
All patients	18,198 (100.0)	1.05 (0.99, 1.11)	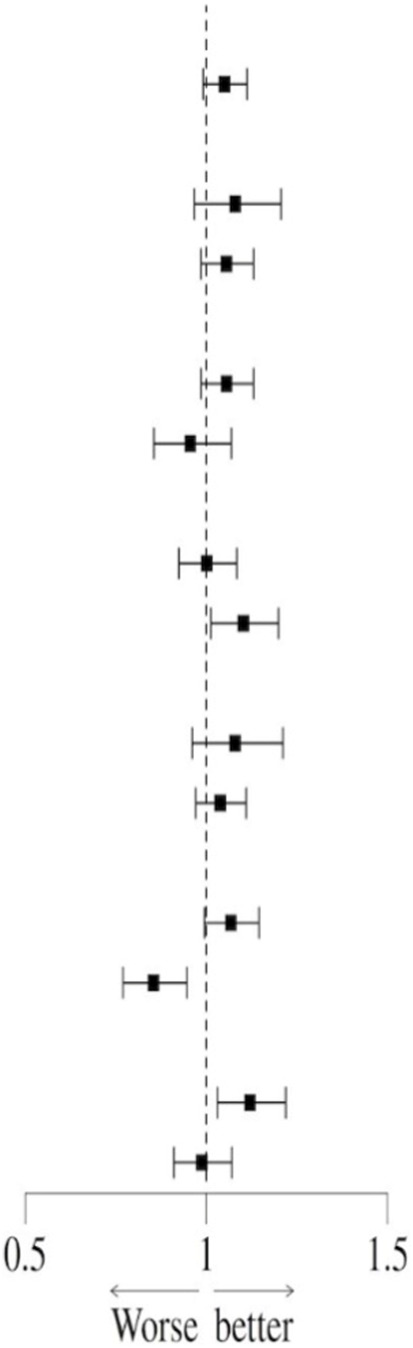	0.100	
Age (yr)					0.517
<65	7,319 (40.2)	1.08 (0.97, 1.21)		0.176	
≥65	10,879 (59.8)	1.06 (0.99, 1.13)		0.122	
ICU LOS days					0.189
<7	14,208 (78.1)	1.06 (0.99, 1.13)		0.122	
≥7	3,990 (21.9)	0.96 (0.85, 1.07)		0.426	
SOFA					0.181
<5	11,277 (62.0)	1.00 (0.92, 1.08)		0.979	
≥5	6,921 (38.0)	1.10 (1.01, 1.20)		0.025	
CCI					0.401
<6	8,148 (44.8)	1.08 (0.96, 1.21)		0.197	
≥6	10,050 (55.2)	1.04 (0.97, 1.11)		0.276	
MV					<.001
<48h	14,264 (78.4)	1.07 (0.99, 1.15)		0.070	
≥48h	3,934 (21.6)	0.85 (0.77, 0.95)		0.003	
Lactate (mmol/L)					0.020
<2	9,913 (54.5)	1.12 (1.03, 1.22)		0.008	
≥2	8,285 (45.5)	0.99 (0.91, 1.07)		0.758	

ICU LOS days, intensive care unit length of stay days; SOFA, sequential organ failure assessment; CCI, Charleson comorbidities index; MV, mechanical ventilation.

## Discussion

This study reveals the complex time effect of PPI on the prognosis of patients with sepsis. Multivariate analysis showed that PPI was an independent risk factor for 90-day all-cause mortality, and the prediction model based on the time series endpoint showed better prognostic recognition efficiency than the traditional scoring system, suggesting that the long-term effects of drug exposure should receive attention in ICU sepsis management. It is worth noting that the survival curve reveals a time-dependent contradiction in survival outcomes, wherein the PPI Users group exhibited a non-significant survival advantage at 28 days (*P* = 0.634) followed by a statistically significant reversal at 90 days (*P* < 0.001). This paradoxical pattern may reflect the biphasic mechanism of PPI, characterized by an early protective effect through reduced stress ulcer risk, whereas prolonged administration could exacerbate adverse outcomes via cumulative disruptions to intestinal microbiota homeostasis or immunomodulatory pathways. Further analysis showed that the severity of disease, duration of mechanical ventilation, and lactic acid metabolism significantly regulated the association between PPI and prognosis, which provide an important basis for individualized medication. In particular, the increased incidence of hypoalbuminemia and opportunistic infections (such as *Clostridium difficile*) in the PPI Users group suggests that protein metabolic disorders and secondary infections may mediate their long-term adverse outcomes. Although prophylactic PPI use may reduce the risk of early gastrointestinal complications, the benefit–risk ratio of continuous medication combined with the results of subgroup analysis need to be carefully evaluated for patients with severe metabolic disorders or long-term mechanical ventilation.

The introduction of Sepsis 3.0 has catalyzed heightened research interest in identifying novel prognostic risk factors and biomarkers associated with sepsis. Nevertheless, the conventional SOFA score may not adequately encapsulate the comprehensive clinical status of the disease, indicating a necessity for refinement. The findings of this study demonstrate that the SOFA score did not achieve statistical significance, and its AUC value was inferior to that of CCI. Furthermore, prior observational studies have indicated that serum albumin may serve as a potential risk factor for mortality among patients with sepsis (Takegawa et al., 2019; Yin et al., 2018). A longitudinal cohort study spanning 17 years has also identified osteoporosis as a novel risk factor for infection and sepsis (Zhang et al., 2022). Additionally, a cohort study utilizing data from MIMIC III has suggested that platelet count may act as an independent predictor of 1-year overall survival in sepsis patients (Zhao et al., 2020). Concurrently, an enhanced scoring system that integrates RDW, age, SOFA, and APACHE II scores—termed the “RAAS score”—has been validated as a reliable tool for the early prediction of short-, medium-, and long-term mortality risks in sepsis patients, reflecting the progressively increasing mortality rates in this population (Huang et al., 2022). These results align with the findings of our research, underscoring the imperative for further investigation into novel biomarkers and scoring systems to enhance prognostic predictions for patients with sepsis.

On the other hand, the utilization and scope of PPI remain subjects of debate within the medical community. Recent guidelines issued by the Society of Critical Care Medicine and the American Society of Health-System Pharmacists advocate for the administration of low-dose PPI or histamine H2 receptor antagonists to all critically ill adults at risk of stress-related upper gastrointestinal bleeding (MacLaren et al., 2024). Finkenstedt et al. (2020) have suggested that PPI should be employed in cases of stress-related mucosal disease (SRMD), acute gastric mucosal lesions, acute erosive gastritis, and acute hemorrhagic gastritis, particularly when multiple risk factors are present. These risk factors can be classified into serious and potential categories. Serious risk factors include MV for more than r 48 h, cardiovascular and cerebrovascular events, chronic liver disease or acute liver failure, coagulation disorders, acute kidney failure or the necessity for renal replacement therapy, severe head and neck spinal cord injuries, shock, persistent hypotension, and sepsis. Potential risk factors include high-dose glucocorticoid therapy, concurrent use of NSAIDs, and prolonged hospital stays exceeding 1 week (Ye et al., 2020). Our investigation revealed low rates of prophylactic PPI use among patients with NSAID consumption and cranial and cervical spinal cord injuries, which contrasts with the findings of Horsa et al. (2019). This inconsistency may stem from the inappropriate prophylactic application of PPI in earlier studies, which could have resulted in a diminished implementation of preventive strategies for at-risk patients. Furthermore, enteral nutrition has been associated with a reduction in the incidence of stress ulcers and the necessity for acid suppression therapy (Barletta, 2023; Jalil and El-Kersh, 2019). The interplay between intestinal clearance and medication use, including PPIs and antibiotics (Weersma et al., 2020), can influence the composition of gut microbiota, modulate immune responses, contribute to small intestinal bacterial overgrowth (Kiecka and Szczepanik, 2023), and elevate the risk of severe infections (Lassalle et al., 2023), such as hospital acquired pneumonia through the “gut lung axis” (Huang et al., 2018; Cotoia et al., 2023). A comprehensive study in the Netherlands has established that PPIs can significantly alter gut microbiota diversity (Bonder et al., 2016).

In recent decades, the application of PPI has been extensively documented across various countries (Liu et al., 2015; Alshamsi et al., 2016). Presently, PPI rank among the ten most commonly prescribed medications, largely due to their favorable side effect profile. Nevertheless, the global understanding of PPI usage remains relatively limited, and concerns regarding the risks and potential adverse effects associated with prolonged PPI therapy have begun to emerge (Fossmark et al., 2019). A systematic review of 28 million PPI users revealed that approximately 25% of adults have utilized these medications, with 63% of users being under the age of 65. Additionally, nearly two-thirds of these individuals were prescribed high doses of PPI (≥defined daily dose), with 25% of users maintaining continuous PPI use for over 1 year and 28% for more than 3 years (Shanika et al., 2023). At present, there is no consensus regarding the preventive application of PPI. The majority of studies suggest that when symptoms are alleviated or risk factors are addressed, a reevaluation of the necessity for continued PPI therapy should be conducted to mitigate potential health risks and reduce treatment costs.

Cheng et al. (2018) categorized patients into three groups based on serum albumin levels: normal (≥35 g/L), marginal hypoalbuminemia (28–34.9 g/L), and hypoalbuminemia (<28 g/L). The findings indicated that all-cause mortality rates increased over time, suggesting that hypoalbuminemia serves as a predictor of mortality and rebleeding in patients experiencing peptic ulcer bleeding who are treated with PPI (Cheng et al., 2018). Furthermore, the use of PPI has been associated with an elevated risk of *C. difficile* infection due to alterations in gut microbiota. Among individuals utilizing PPI, there was a significant increase in bacterial populations, including *Enterococcus*, *Streptococcus*, *Staphylococcus*, and potential pathogen *Escherichia coli* (Imhann et al., 2016). In addition to the adverse effects identified in this study, a systematic review and meta-analysis revealed that frequent and prolonged use of PPI is linked to various adverse outcomes, including gastric cancer, micronutrient deficiencies (such as magnesium and iron), acid rebound, infections, fractures, dementia, kidney disease (particularly in elderly patients with pre-existing renal conditions), sudden death, cardiovascular changes (including MI), and pneumonia (Chinzon et al., 2022). These potential adverse reactions carry substantial clinical implications and necessitate further investigation.

This study acknowledges several limitations. First, its retrospective single-center design limited causal inference between PPI use and sepsis compared with prospective studies. Second, data sourced from the MIMIC database may introduce variability in sepsis diagnostic criteria and PPI management protocols across regions, necessitating external validation. Finally, suboptimal variable screening methodologies with residual adaptive bias risks, coupled with insufficient subgroup analyses (e.g., enteral nutrition timing, formula selection, PPI dosing, and treatment duration), highlight the imperative for model refinement through targeted research.

## Conclusion

This study revealed that prophylactic PPI use was associated with increased 90-day all-cause mortality in ICU patients with sepsis, although it showed no significant effect on 28-day mortality. While prophylactic PPI use might shorten ICU length of stay, it did not improve 28- or 90-day survival rates nor reduce total hospital stay duration. Additionally, PPI use may elevate the risk of hypoalbuminemia and *Clostridioides difficile* infection. These findings suggest that clinicians should carefully weigh the potential benefits against adverse effects of PPI prophylaxis. Future prospective studies are warranted to clarify the mechanisms underlying its long-term prognostic impact.

## Data Availability

The datasets presented in this study can be found in online repositories. The names of the repository/repositories and accession number(s) can be found in the article/Supplementary Material.

## Glossary

AKI: Acute kidney injury
APTT: Activated partial thromboplastin time
BIDMC: Beth Israel Deaconess Medical Center
BMI: Body mass index
BUN: Blood urea nitrogen
CCI: Charlson comorbidities index
CHF: Congestive heart failure
CI: Confidence interval
COPD: Chronic obstructive pulmonary disease
DC: Decision curve analysis
ECMO: Extracorporeal membrane oxygenation
GI: Gastrointestinal bleeding
Hospital LOS days: Hospital length of stay days
HR: Hazard ratio
ICU: Intensive care unit
ICU LOS days: Intensive care unit length of stay days
INR: International standard ratio
IV: Intravenous injection or other intravenous routes of administration
L%: Percentage of lymphocytes
MAP: Mean arterial pressure
**Mg** ^**2+**^: Magnesium
MI: Myocardial infarction
MIMIC-IV 2.2: Medical Information Mart for Intensive Care IV
MV: Mechanical ventilation
N%: Percentage of neutrophils
NSAIDs: Nonsteroidal anti-inflammatory drugs
**PCO**_**2**_: Partial pressure of carbon dioxide
PLT: Platelet count
PO: Per os
**PO**_**2**_: Partial pressure of oxygen
PPIs: Proton pump inhibitors
PT: Prothrombin time
ROC: Receiver operating characteristic
RR: Relative risk
RRT: Renal replacement therapy
SAPA II: Simplified acute physiology score
SMD: Standardized mean difference
SOFA: Sequential organ failure assessment
**SpO**_**2**_: Pulse oxygen saturation
SRMD: Stress-related mucosal disease
TUBE: Gastrostomy tube
WBC: White blood cell
